# Energy Minimisers with Prescribed Jacobian

**DOI:** 10.1007/s00205-021-01699-4

**Published:** 2021-10-14

**Authors:** André Guerra, Lukas Koch, Sauli Lindberg

**Affiliations:** 1 University of Oxford, Andrew Wiles Building Woodstock Rd, Oxford, OX2 6GG UK; 2 Department of Mathematics and Statistics, University of Helsinki, P.O. Box 68, 00014 Helsingin Yliopisto, Finland

## Abstract

We consider the class of planar maps with Jacobian prescribed to be a fixed radially symmetric function *f* and which, moreover, fixes the boundary of a ball; we then study maps which minimise the 2*p*-Dirichlet energy in this class. We find a quantity $\lambda [f]$ which controls the symmetry, uniqueness and regularity of minimisers: if $\lambda [f]\le 1$ then minimisers are symmetric and unique; if $\lambda [f]$ is large but finite then there may be uncountably many minimisers, none of which is symmetric, although all of them have optimal regularity; if $\lambda [f]$ is infinite then generically minimisers have lower regularity. In particular, this result gives a negative answer to a question of Hélein (Ann. Inst. H. Poincaré Anal. Non Linéaire 11(3):275–296, 1994). Some of our results also extend to the setting where the ball is replaced by ${\mathbb {R}}^2$ and boundary conditions are not prescribed.

## Introduction

Given a domain $\varOmega \subset {\mathbb {R}}^n$, an orientation-preserving diffeomorphism $u_0:{{\overline{\varOmega }}}\rightarrow u_0({{\overline{\varOmega }}})$ and a continuous stored-energy function ${W:\overline{\varOmega }\times \text{ GL}^+(n)\rightarrow {\mathbb {R}}}$, a typical problem in nonlinear elastostatics is to

1.1 $$\begin{aligned} \text{ minimise } {\mathscr {W}}[u]\equiv \int _\varOmega W(x,\mathrm{D}u)\,\mathrm{d}x, \quad \text{ among } \text{ all } u\in u_0+W_0^{1,\infty }(\varOmega ,{\mathbb {R}}^n); \nonumber \\ \end{aligned}$$

see for instance [2, 3]. In order for the Direct Method to be applicable, ${\mathscr {W}}$ needs to sequentially weakly lower semicontinuous and, more importantly for our discussion here, coercive [17]. However, one is sometimes led to consider non-coercive energy functions and this is the case, for instance, when *W* depends on $\mathrm{D}u$ only through $\mathrm{J}u\equiv \det \mathrm{D}u$, see e.g. [18, 32, 34] for examples in the study of elastic crystals and [22] for a different example. In this case, a solution of (1.1) is found, at least formally, by solving the following problem:

$$\begin{aligned} {\left\{ \begin{array}{ll} \mathrm{J}u = f &{} \text{ in } \varOmega ,\\ u=u_0 &{} \text{ on } \partial \varOmega , \end{array}\right. } \qquad \text{ where } f \text{ is } \text{ such } \text{ that } W(x,f(x))\equiv \min _{\xi \hbox {>} 0} W(x,\xi ). \end{aligned}$$

We thus see that the existence and regularity of solutions to (1.1) can be approached by studying the existence and regularity of solutions to the prescribed Jacobian equation

1.2 $$\begin{aligned} \mathrm{J}u \equiv \det \mathrm{D}u= f \quad \text{ in } \varOmega . \end{aligned}$$

In this paper we will focus on the prescribed Jacobian Equation (1.2) and, for now, we will assume that $f\in L^1(\varOmega )$ is positive. If *u* is an orientation-preserving diffeomorphism, the prescribed Jacobian Equation (1.2) is the differential analogue of the integral relation

1.3 $$\begin{aligned} {\mathscr {L}}^n(u(E))=\int _E f \,\mathrm{d}x, \end{aligned}$$

which expresses the fact that *u* transports the measure $f \,\mathrm{d}x$ to a measure with uniform density. The existence of homeomorphisms *u* satisfying (1.3) is very classical, see for instance [1] for an overview of the theory. The regularity question is then natural: given *f*, how regular can the transport map *u* be? This is a *transport problem without cost concerns*: we are only interested in finding a transport map as smooth as possible.

If *f* is Hölder-continuous or smoother then one can find transport maps with optimal regularity and, in this setting, there is a rich well-posedness theory [21] which goes back to the works of Moser and Dacorogna–Moser [23, 53]. Counter-examples to well-posedness for data which is merely continuous were obtained in [15, 52]. Moreover, very little is known in the low regularity setting when *f* is just an $L^p$ function, but we refer the reader to [31, 40, 48, 51, 56] for results in this direction.

In this paper, we continue the program initiated in our recent works [36, 37] and we study (1.2) for $f\in L^p(\varOmega )$. Apart from the nonlinear character of the Jacobian, the main obstacle in studying existence and regularity of solutions to (1.2) is the *underdetermined* nature of the equation, as transport maps are far from unique. In particular, it is highly unclear how to select transport maps with optimal regularity.

A natural selection criterion, often used in Optimal Transport [14, 24], is to consider maps which minimise the quadratic cost

$$\begin{aligned} {\mathscr {C}}[u]\equiv \int _{\varOmega }\frac{|u(x)-x|^2}{2}\,f(x) \,\mathrm{d}x. \end{aligned}$$

However, and while ${\mathscr {C}}$ does single out a unique solution of (1.2), one can sometimes find other solutions of (1.2) with better regularity; see [59, p. 293] as well as [37, p. 2]. In fact, the same phenomenon was observed by Bourgain and Brezis in [12] for the divergence equation, which one can regard as the linear counterpart of the Jacobian Eq. (1.2). In conclusion, underdetermined equations often admit solutions which have a surprising amount of regularity.

As we are interested in Sobolev regularity of solutions to (1.2), in this paper we will investigate whether minimisation of the *np*-Dirichlet energy is an appropriate selection criterion.

### Definition 1.1

For $p\ge 1$ and $f\in L^1_\text{ loc }(\varOmega )$, we define the *np*-*energy of* *f* as

$$\begin{aligned} {\mathscr {E}}_{np}(f,\varOmega )\equiv \inf \left\{ \int _{\varOmega }|D v|^{np}\,d x : v\in {\dot{W}}^{1,np}(\varOmega ,{\mathbb {R}}^n) \text { satisfies } J v = f \text { a.e. } \text { in } \varOmega \right\} . \end{aligned}$$

Given $f\in L^1_\text{ loc }(\varOmega )$, we say that $u\in {\dot{W}}^{1,np}(\varOmega , {\mathbb {R}}^n)$ is a *np*-*energy minimiser for* *f* if

$$\begin{aligned} {\mathscr {E}}_{np}(f,\varOmega ) = \int _{\varOmega } |D u|^{np}\,d x \qquad \text { and } \qquad J u = f \text { a.e. } \text { in } \varOmega . \end{aligned}$$

In Definition 1.1, as in the rest of the paper, |*A*| denotes the Hilbert–Schmidt norm of a matrix $A\in {\mathbb {R}}^{n\times n}$. Note that, from the Direct Method and the weak continuity of the Jacobian, it follows that if (1.2) admits a solution in ${\dot{W}^{1,np}}$, then there is at least one *np*-energy minimiser.

A parallel question, which we will also discuss, concerns the regularity of energy minimal solutions. Establishing regularity of energy minimisers for (1.2) is a challenging task, even in the incompressible case $f=1$. There is an extensive literature on the topic, and we refer the reader to [7, 10, 16, 27, 45, 46], as well as the references therein, for further information.

In Section 1.1 we will discuss the Dirichlet problem for (1.2), while in Sect. 1.2 we study (1.2) in the important case where $\varOmega ={\mathbb {R}}^n$.

### Energy Minimisers for the Dirichlet Problem

In this subsection we consider the Dirichlet problem for (1.2) over the unit ball $B\subset {\mathbb {R}}^n$:

1.4 $$\begin{aligned} {\left\{ \begin{array}{ll} \mathrm{J}u = f &{} \text{ a.e. } \text{ in } B,\\ u=\text{ id } &{} \text{ on } \partial B. \end{array}\right. } \end{aligned}$$

We assume that *f* is compatible with the boundary condition, as well as uniformly positive:

1.5 $$\begin{aligned} \int _B f \,\mathrm{d}x = 1, \qquad \mathop {\hbox {ess inf}}\limits _B f\ge c>0, \end{aligned}$$

where *c* is some fixed constant. As in [37], we also introduce the complete metric spaces

$$\begin{aligned} X^p(B)\equiv {\left\{ \begin{array}{ll} \{f\in L^p(B):(1.5) \text{ holds }\} &{} \text{ if } p>1,\\ \{f\in L\log L(B):(1.5) \text{ holds }\} &{} \text{ if } p=1. \end{array}\right. } \end{aligned}$$

The particular definition of $X^1(B)$ is due to the improved integrability of the Jacobian: the local $L\log L$ integrability of non-negative Jacobians of $W^{1,n}$ maps was proved by Müller in [54], and a global version, under suitable boundary regularity, was proved in [39].

We will focus on the case $n=2$, as it already exhibits the main challenges of the problem. One of the main advantages of taking $n=2$ is that, by the Iwaniec–Šverák theory of mappings of integrable distortion [43], under assumptions (1.5), a map $u\in W^{1,2}(B,{\mathbb {R}}^2)$ solving (1.4) is necessarily a homeomorphism $u:{{\overline{B}}} \rightarrow {{\overline{B}}}$, see for instance [37] for further details. In higher dimensions, the same conclusion is true only for maps whose derivatives have higher integrability.

Suppose that $f\in X^p(B)$ is radially symmetric, i.e. $f=f(|z|)$. In this case, there is a unique radial stretching, denoted $\phi _1$, solving (1.4):

1.6 $$\begin{aligned} \phi _1(z)\equiv \rho (|z|) \frac{z}{|z|}, \qquad \text{ where } \rho (r) \equiv \sqrt{\int _0^r 2 s f(s) \,\mathrm{d}s}. \end{aligned}$$

Here the subscript ‘1’ denotes the topological degree of the map, see Definition 2.3 for more general solutions. Naturally we would like to relate the regularity of $\phi _1$ with that of *f*. It turns out that the relevant quantity here is

1.7 $$\begin{aligned} \lambda [f]\equiv \mathop {\hbox {ess sup}}\limits _{r\in [0,1]}\frac{|f(r)|}{\fint _{B_r(0)} f \,\mathrm{d}x}. \end{aligned}$$

Indeed, we have

#### Proposition A

(Regularity of symmetric solutions) Let $1\le p<\infty$ and take a radially symmetric $f\in X^p(B)$. If $\lambda [f]<\infty$ then we have the estimate

$$\begin{aligned} \Vert D \phi _1\Vert ^2_{L^{2p}(B)} \lesssim (1+\lambda [f]^2) \,\Vert f\Vert _{X^p(B)}. \end{aligned}$$

However, in general $\lambda [f]=+\infty$ and $D \phi _1\in L^p\backslash L^{p+\varepsilon }(B)$ for any $\varepsilon >0$.

Due to the underdetermined nature of (1.4), it is not clear whether, when $\lambda [f]=+\infty$, one can find solutions with better regularity than the symmetric ones. In [37] we showed that, for a certain class of data which is in $L^p\backslash L^{p+\varepsilon }$ near the boundary, the symmetric solutions are energy quasiminimisers, i.e. they have energy comparable to that of the energy minimal solutions. It follows that sometimes the symmetric solutions have optimal regularity even when $\lambda [f]=+\infty$.

It is then natural to ask whether the symmetric solutions have minimal energy, so we have

#### Question 1.2

(Hélein) Let *f* be radially symmetric. Is the unique radial stretching $\phi _1$ solving (1.4) a 2-energy^1^ minimiser for *f* in the class $W^{1,2}_\text{ id }(B,B)$?

The main result of this paper shows that $\lambda [f]$ controls not only the regularity of the symmetric solutions, but also the uniqueness and symmetry of energy minimisers for *f*.

#### Theorem B

(Symmetry versus symmetry breaking) Let $1\le p <\infty$ and $f\in X^p(B)$ be radially symmetric. We have two regimes.

(i) if $\lambda [f]\le 1$ then $\phi _1$ is the unique 2*p*-energy minimiser for *f* in $W^{1,2p}_ id (B,{\mathbb {R}}^2)$;
(ii) if $\lambda [f]\gg 1$ then there may be non-symmetric energy minimisers. More precisely, for any $p_0\in (1,\infty )$, there is $f\in X^\infty (B)$ such that, for any $p\in [1,p_0]$, there are uncountably many 2*p*-energy minimisers for *f* in $W^{1,2p}_id (B,B)$, none of which is symmetric.

Theorem B shows that energy minimisation is not a suitable selection criterion and that, in general, the answer to Question 1.2 is negative. We now make several remarks concerning Theorem B.

#### Remark 1.3

(Boundary condition) The proof of Theorem B(i) does not require the boundary condition $u=\text{ id }$ on $\partial B$. In fact, we prove the following stronger result. Let us write

1.8 $$\begin{aligned} \lambda _R[f]\equiv \mathop {\hbox {ess sup}}\limits _{r\in [0,R]}\frac{|f(r)|}{\fint _{B_r(0)} f \,\mathrm{d}x}. \end{aligned}$$

If $\mathrm{J}u=f$ in $B_R(0)$ and $\lambda _R[f] \le 1$, then

$$\begin{aligned} \Vert \mathrm{D}\phi _1\Vert _{L^{2p}(B_R(0))} \le \Vert \mathrm{D}u\Vert _{L^{2p}(B_R(0))} \end{aligned}$$

with equality if and only if, in $B_R(0)$, $u=e^{i \alpha } \phi _{\pm 1}+z_0$, for some $\alpha \in [0,2\pi ]$ and $z_0\in {\mathbb {C}}$.

#### Remark 1.4

(Transition between regimes) Let $\varLambda =\varLambda (p)$ be the largest number such that

$$\begin{aligned} \lambda [f]\le \varLambda \quad \implies \phi _1 \text{ is } \text{ the } \text{ unique } 2p\text{-energy } \text{ minimiser } \text{ in } W^{1,2p}_\text{ id }(B,{\mathbb {R}}^2). \end{aligned}$$

Theorem B shows that $\varLambda \in [1,+\infty )$. We do not know whether $\varLambda >1$. Moreover, as the proof of Theorem B is perturbative, we do not have explicit upper bounds on $\varLambda$.

#### Remark 1.5

(Symmetry versus uniqueness) When *f* is radially symmetric, problem (1.4) has a 1-dimensional group of symmetries

$$\begin{aligned}{}[0,2\pi ] \ni \alpha \mapsto u_\alpha \equiv e^{-i \alpha } u(e^{i\alpha }\cdot ); \end{aligned}$$

in particular, if *u* is a solution of (1.2), so is $u_\alpha$. Moreover, *u* and $u_\alpha$ have the same energy. An energy minimiser $u\in W^{1,2}_\text{ id }$ is preserved by this group of symmetries, i.e. $u_\alpha = u$ for all $\alpha \in [0,2\pi ],$ if and only if *u* is a radial stretching, c.f. Lemma 2.8 below. It follows that, for a given symmetric data, there is a unique energy minimiser in $W^{1,2p}_\text{ id }(B,{\mathbb {R}}^n)$ if and only if the symmetric solution has minimal energy; if this is not the case, then necessarily one has at least a 1-dimensional manifold of minimisers.

Let us now explain the role played by $\lambda [f]$ in controlling the symmetry of minimisers. Writing $z=r e^{i \theta }$, note that

$$\begin{aligned} |\mathrm{D}u|^2 = |\partial _r u|^2 + \frac{|\partial _\theta u|^2}{r^2} , \qquad \mathrm{J}u= \frac{\partial _\theta u}{r} \wedge \partial _r u . \end{aligned}$$

Hence we see that energy minimisation favours maps for which

(i) $\partial _r u$ is approximately perpendicular to $\partial _\theta u$, so that $|\mathrm{J}u|\approx |\partial _r u||\partial _\theta u|/r$;
(ii) $|\partial _r u|\approx \frac{1}{r} |\partial _\theta u|$, so that $|\partial _r u |\frac{|\partial _\theta u|}{r}\approx \frac{1}{2}(|\partial _r u |^2+|\partial _\theta u |^2/r^2)=\frac{1}{2}|\mathrm{D}u|^2$.

Recalling (1.6), we see that radial stretchings accomplish (i) perfectly, indeed,

1.9 $$\begin{aligned} \mathrm{D}\phi _1(z) = \frac{\rho (r)}{r} \text{ Id } + \bigg ( {{\dot{\rho }}}(r)-\frac{\rho (r)}{r}\bigg )\frac{z\otimes z}{r^2}\quad \implies \quad {\left\{ \begin{array}{ll} \partial _r \phi _1 = {{\dot{\rho }}}(r) z, \\ \frac{1}{r} \partial _\theta \phi _1 = \frac{\rho (r)}{r} z^\bot . \end{array}\right. } \end{aligned}$$

There is, however, no reason for radial stretchings to satisfy (ii), and this is where the condition $\lambda [f]\le 1$ comes in: we have $\lambda [f]\le 1$ if and only if

1.10 $$\begin{aligned} |\partial _r \phi _1(x)| \le \frac{1}{r} |\partial _\theta \phi _1(x)| \text{ for } \text{ a.e. } x \text{ in } B, \end{aligned}$$

c.f. Lemma 2.6. The isoperimetric inequality shows that $\phi _1$ has optimal angular derivatives among all solutions of (1.2), while $\lambda [f]\le 1$ ensures that these derivatives control the Dirichlet energy $|\mathrm{D}\phi _1|^2$. Through elementary linear algebra and a convexity argument we are then able to prove that $\phi _1$ is an energy minimiser.

A simple sufficient condition on *f* which guarantees that $\lambda [f]\le 1$ is that $r\mapsto f(r)$ is non-increasing. We also note that condition (1.10) is not new: a radial stretching satisfying (1.10) was called *conformally non-expanding* in [42], as it does not increase the conformal modulus of annuli.

### Energy Minimisers in ${\mathbb {R}}^n$

In this subsection we study (1.2) over ${\mathbb {R}}^n$. In this setting, the following question, essentially set by Coifman, Lions, Meyer and Semmes in [19], remains an outstanding open problem:

#### Question 1.6

Is the Jacobian $\mathrm{J}:{\dot{W}}^{1,np}({\mathbb {R}}^n,{\mathbb {R}}^n)\rightarrow \mathscr {H}^p({\mathbb {R}}^n)$ surjective?

Here ${\mathscr {H}}^p({\mathbb {R}}^n)$ stands for the real Hardy space and we refer the reader to [20, 58] for its theory. We recall that, for $p>1$, ${\mathscr {H}}^p({\mathbb {R}}^n)$ agrees with the usual Lebesgue space $L^p({\mathbb {R}}^n)$.

Question 1.6 is especially natural as, in [19, 40], it is shown that $\mathscr {H}^p({\mathbb {R}}^n)$ is the smallest Banach space containing the range of the Jacobian; compare with [51] for the *inhomogeneous* case. In [41] (see also [11]), Iwaniec went further than Question 1.6 by posing the following:

#### Conjecture 1.7

For each $p\in [1,\infty )$, the Jacobian has a *continuous right inverse*: there is a continuous map $E:{\mathscr {H}}^p({\mathbb {R}}^n)\rightarrow {\dot{W}}^{1,np}({\mathbb {R}}^n,{\mathbb {R}}^n)$ such that $J \circ E = Id$.

Let us say that two maps $u,v:{\mathbb {R}}^n\rightarrow {\mathbb {R}}^n$ are *equal modulo rotations* if there is $Q\in \text{ SO }(n)$ such that $u(x)=Q v(x)$ for a.e. *x*; in this case, if $\mathrm{J}u=f$ in ${\mathbb {R}}^n$, then $\mathrm{J}v=f$ in ${\mathbb {R}}^n$ as well. In [41], Iwaniec proposed the following route towards Conjecture 1.7:

#### Strategy 1.8

A possible way of proving Conjecture 1.7 is to establish the following claims:

(i) Every *np*-energy minimiser satisfies $\Vert \mathrm{D}u \Vert ^n_{L^\mathrm{np}({\mathbb {R}}^n)} \lesssim \Vert \mathrm{J} u \Vert _{{\mathscr {H}}^p({\mathbb {R}}^n)}$.
(ii) For all $f \in {\mathscr {H}}^p({\mathbb {R}}^n)$ there is a unique *np*-energy minimiser $u_f$ for *f*, modulo rotations.
(iii) For all $f\in {\mathscr {H}}^p({\mathbb {R}}^n)$ there is a rotation $Q_f\in \text{ SO }(n)$ such that $f\mapsto Q_f u_f$ is continuous.

The nonlinear open mapping principles that we proved in [36] show that (i) is *equivalent* to a positive answer to Question 1.6. In this direction, Iwaniec has suggested that one should prove (i) by constructing a Lagrange multiplier for every *np*-energy minimiser, see the third author’s works [49, 50] for results in this direction.

Using the terminology of [36], we say that a solution in ${\dot{W}}^{1,n}({\mathbb {R}}^n,{\mathbb {R}}^n)$ of (1.2) is *admissible* if it is continuous and it satisfies the change of variables formula, i.e.,

$$\begin{aligned} \int _{{\mathbb {R}}^n} \#\{x\in E: u(x)=y\}\,\mathrm{d}y= \int _E f \,\mathrm{d}x ; \end{aligned}$$

this is the generalization of (1.3) to maps which are not necessarily injective. We note that solutions in ${\dot{W}}^{1,np}({\mathbb {R}}^n,{\mathbb {R}}^n)$, for $p>1$, are always admissible, c.f. Remark 3.4. Due to Remark 1.3, the proof of Theorem B(i) can be adapted to ${\mathbb {R}}^2$, and it provides conditions on *f* under which claims (i) and (ii) of Strategy 1.8 hold:

#### Corollary C

Fix $1\le p <\infty$. Let $f\in {\mathscr {H}}^p({\mathbb {R}}^2)$ be a radially symmetric function such that

$$\begin{aligned} |f(r)|\le \fint _{B_r(0)} f \,\mathrm{d}x\qquad \text{ for } \text{ a.e. } r\in (0,\infty ). \end{aligned}$$

Then $\Vert \mathrm{D}\phi _1 \Vert ^2_{L^{2p}({\mathbb {R}}^2)} \lesssim \Vert f \Vert _{{\mathscr {H}}^p({\mathbb {R}}^2)}$ and, for $p>1$, $\phi _1$ is the unique 2*p*-energy minimiser for *f*, modulo rotations. For $p=1$ the same statement holds in the class of admissible solutions.

Further uniqueness results can be found in [49]. Nonetheless, in light of Theorem B(ii) one expects that, in general, energy minimisers are not unique, even for radially symmetric data. As the final result of this paper we show that this is indeed the case, and hence claim (ii) in Strategy 1.8 is false.

#### Theorem D

(Non-uniqueness) Fix $1\le p<\infty$. There exists a $C^1$ radially symmetric function ${f\in {\mathscr {H}}^p({\mathbb {R}}^2)}$ which has uncountably many 2*p*-energy minimisers, modulo rotations.

Thus, in this setting, energy minimisation is once again not a suitable selection criterion. Moreover, it is very difficult to work with energy minimisers directly: when $p=1$, we cannot decide whether they are admissible, although in [36] we showed that, under natural assumptions, the existence of energy minimisers implies the existence of admissible solutions.

To conclude the discussion of Strategy 1.8, we note that, assuming that (i) holds, it remains to establish a nonlinear analogue of the classical Bartle–Graves theorem [6]. This theorem states that a bounded linear surjection between Banach spaces has a bounded and continuous (but possibly nonlinear) right inverse, see [8, page 86] for a good overview. Without extra assumptions, the Bartle–Graves theorem does not generalise to multilinear mappings [30]. However, one may use the results in [36] and [44] to prove a partial result towards a nonlinear Bartle–Graves theorem for the Jacobian: assuming surjectivity of $\text { J}:{\dot{W}}^{1,np}({\mathbb {R}}^n,{\mathbb {R}}^n) \rightarrow {\mathscr {H}}^p({\mathbb {R}}^n)$, there is a bounded right inverse that is continuous outside a meagre set, although we do not prove such a result here.

### Notation

We use polar coordinates $z=r e^{i \theta }= x + iy \in {\mathbb {C}}\cong {\mathbb {R}}^2$ in the plane. We write $B_r(x)$ for the usual Euclidean balls in ${\mathbb {R}}^n$, and $\mathbb S_r\equiv \partial B_r$ (when *x* is omitted, it is understood that $x=0$). It is also useful to have notation for annuli: for $0<r<R$,

$$\begin{aligned} \mathbb A(r,R)\equiv \{z\in {\mathbb {C}}: r<|z|<R\}. \end{aligned}$$

We will also abuse this notation slightly by setting $\mathbb A(0,R)\equiv B_R(0)$. Here |*z*| denotes the Euclidean norm of $z\in {\mathbb {C}}$ and likewise for $A\in {\mathbb {R}}^{n\times n}$ we write $|A|\equiv \text{ tr }(A A^\text{ T})^\frac{1}{2}$ for the Euclidean norm. Finally, and unless stated otherwise, *p* is a real number in the interval $[1,+\infty )$.

### Outline

This paper is structured as follows: in Sect. 2 we consider the regularity of polar representations of a Sobolev map and we recall some useful formulae in polar coordinates; we also prove Proposition A. In Sect. 3 we prove a more general version of Theorem B(i), as well as Corollary C. In Sect. 4 we prove Theorem B(ii). Finally, Sect. 5 contains the proof of Theorem D.

## Polar Coordinates and Generalised Radial Stretchings

Given a planar Sobolev map $u\in W^{1,p}({\mathbb {R}}^2,{\mathbb {R}}^2)$, we consider polar coordinates both in the domain and in the target; that is, we want to write

2.1 $$\begin{aligned} u(r e^{i \theta }) = \psi (r,\theta ) \exp ( i \gamma (r,\theta )) \end{aligned}$$

for some functions $\psi :(0,\infty )\times [0,2\pi ]\rightarrow [0,\infty )$ and $\gamma :(0,\infty )\times [0,2\pi ] \rightarrow {\mathbb {R}}$, where furthermore we must have the compatibility conditions

2.2 $$\begin{aligned} \psi (r,0)=\psi (r,2\pi ) \quad \text{ and } \quad \gamma (r,0)-\gamma (r,2\pi )\in 2\pi {\mathbb {Z}}\qquad \text{ for } \text{ all } r. \end{aligned}$$

We will freely identify $(r,\theta )\equiv r e^{i \theta }$, adopting either notation whenever it is more convenient.

The existence of a representation as in (2.1) is a standard problem in lifting theory.

### Proposition 2.1

Let $0\le R_1<R_2$ and $p\ge 2$. Let $u\in W^{1,p}(\mathbb A(R_1,R_2),{\mathbb {R}}^2)$ and, if $p=2$, suppose moreover that *u* is continuous. Assume $0\not \in u(\mathbb A(R_1,R_2))$. Then there are continuous functions

$$\begin{aligned} \psi \in W^{1,p}\left( [R_1,R_2]\times [0,2\pi ]\right) , \qquad \gamma \in W^{1,p}\left( (\max \{R_1,\varepsilon \},R_2)\times [0,2\pi ]\right) , \end{aligned}$$

where $\varepsilon \in (0,R_2)$ is arbitrary, which satisfy (2.2) and such that the representation (2.1) holds.

We remark that the conclusion of Proposition 2.1 is false if $p<2$; see [13, §4].

### Proof

Let $\varepsilon >0$ and consider the keyhole domains

$$\begin{aligned}&\mathbb A_{1,\varepsilon } \equiv [\max \{R_1,\varepsilon \},R_2]\times [\varepsilon ,2\pi -\varepsilon ],\\&\mathbb A_{2,\varepsilon } \equiv [\max (R_1,\varepsilon ),R_2]\times ([0,\pi -\varepsilon ]\cup [\pi +\varepsilon ,2\pi ]). \end{aligned}$$

We freely identify $\mathbb A_{i,\varepsilon }$ with the respective domains in ${\mathbb {R}}^2$.

We first show the existence of a representation (2.1) in each $\mathbb A_{i,\varepsilon }$. Note that if $u\in W^{1,p}$ then $\psi =|u|$ is also in $W^{1,p}$ and is continuous whenever *u* is. Thus, since $0\not \in u(\mathbb A_{i,\varepsilon })$, it suffices to prove the existence of a continuous function $\gamma _i\in W^{1,p}(\mathbb A_{i,\varepsilon },{\mathbb {R}})$ such that $u/|u|= e^{i \gamma _i}$ for $i=1,2$. Since *u* is continuous, $u/|u|\in W^{1,p}(\mathbb A_{i,\varepsilon },\mathbb S^1)$, and so the existence of $\gamma _i$ follows from the results in [9]; see also [13].

Thus, for almost every $(r,\theta )\in \mathbb A_{1,\varepsilon }\cap \mathbb A_{2,\varepsilon }$,

$$\begin{aligned}&\psi (r,\theta )e^{i\gamma _1(r,\theta )}=u(r e^{i \theta })\\&\quad =\psi (r,\theta ) e^{i\gamma _2(r,\theta )}\iff \gamma _1(r,\theta )-\gamma _2(r,\theta ) = 2\pi k(r,\theta ), \end{aligned}$$

where $k(r,\theta )\in {\mathbb {Z}}$. As $\gamma _1$, $\gamma _2$ are continuous in $\mathbb A_{1,\varepsilon }\cap \mathbb A_{2,\varepsilon }$, we must have

$$\begin{aligned}k(r,\theta )= {\left\{ \begin{array}{ll} k_1 \qquad \text { for } \varepsilon<\theta<\pi -\varepsilon ,\\ k_2 \qquad \text { for } \pi +\varepsilon<\theta <\varepsilon . \end{array}\right. } \end{aligned}$$

Without loss of generality, upon redefining $\gamma _1$, we may assume that $k_1=0$. Hence we may define

$$\begin{aligned} \gamma _\varepsilon (r,\theta )={\left\{ \begin{array}{ll} \gamma _1(r,\theta ) &{}\text { if } (r,\theta )\in \mathbb A_{1,\varepsilon },\\ \gamma _2(r,\theta ) &{}\text { if } (r,\theta )\in \mathbb A_{2,\varepsilon }. \end{array}\right. } \end{aligned}$$

By a similar argument, we can ensure that ${\gamma _\varepsilon =\gamma _\delta }$ in ${\mathbb A(R_1,R_2){\setminus } (B_\delta \cup B_\varepsilon )}$, so that in fact

$$\begin{aligned} u=\psi (r,\theta )e^{i\gamma (r,\theta )} \end{aligned}$$

with $\gamma \in W^{1,p}(\max (R_1,\varepsilon ),R_2)\times [0,2\pi ])$ for all $\varepsilon >0$. The conclusion follows.

$$\square $$

### Corollary 2.2

In the setting of Proposition 2.1, we have a.e. the formulae

2.3 $$\begin{aligned} \mathrm{J} u= & {} \frac{\psi }{r}\left( \partial _r\psi \,\partial _\theta \gamma -\partial _\theta \psi \partial _r \gamma \right) , \end{aligned}$$

2.4 $$\begin{aligned} |\mathrm{D}u|^2= & {} |\partial _r \psi |^2+|\psi \partial _r \gamma |^2 +\frac{|\partial _\theta \psi |^2}{r^2} +\frac{|\psi \partial _\theta \gamma |^2 }{r^2}. \end{aligned}$$

### Proof

It is not difficult to formally derive the above formulae whenever the representation (2.1) holds. To make the argument rigorous it suffices to note that, due to the regularity of $\psi$ and $\gamma$, the right-hand sides in (2.3)–(2.4) define locally integrable functions. Thus the corollary follows by a standard density argument.

$$\square $$

A function $f:B_R(0)\rightarrow {\mathbb {R}}$ is said to be *radially symmetric* if it holds that

$$\begin{aligned} {|x|=|y|\implies f(x)=f(y)}, \end{aligned}$$

and we identify any such function with a function $f:[0,+\infty )\rightarrow {\mathbb {R}}$ in the obvious way. For such a function, it is natural to look for solutions of (1.2) possessing some symmetry, in particular satisfying $\partial _\theta \psi =0$ and $\partial _r \gamma = 0$ if a representation as in (2.1) holds:

### Definition 2.3

The class of *generalised radial stretchings* consists of maps of the form

$$\begin{aligned} \phi _k(z)\equiv \frac{\rho (r)}{\sqrt{|k|}} e^{i k \theta }, \end{aligned}$$

where $k\in {\mathbb {Z}}{\setminus }\{0\}$ is the topological degree of the map and $\rho :[0,+\infty )\rightarrow [0,+\infty )$. If $k=1$ we refer to such maps simply as *radial stretchings*.

Generalised radial stretchings are spherically symmetric in the sense that they map circles centred at zero to circles centred at zero. The following lemma gives a useful criterion concerning the Sobolev regularity of generalised radial stretchings:

### Lemma 2.4

Let $p\in [1,\infty )$ and $k\in {\mathbb {Z}}{\setminus }\{0\}$. Given $R\in (0,+\infty ]$, we have that ${\phi _k\in {\dot{W}}^{1,p}(B_R(0), B_R(0))}$ if and only if $\rho$ is absolutely continuous on (0, *R*) and

$$\begin{aligned} \Vert \mathrm{D}\phi _k \Vert _{L^p(B_R(0))}^p\approx \int _0^R \left( \bigg |\frac{{{\dot{\rho }}}(r)}{\sqrt{|k|}}\bigg |^p + \bigg |\sqrt{|k|}\, \frac{\rho (r)}{r}\bigg |^p \right) r \mathrm{d} r<\infty . \end{aligned}$$

We omit the proof of the lemma, as it is a straightforward adaptation of [4, Lemma 4.1].

It is not the case that any radially symmetric $f\in \mathscr {H}^p({\mathbb {R}}^2)$ admits generalised radial stretchings as solutions of (1.2). Indeed, from (2.3), formally we see that

2.5 $$\begin{aligned} \mathrm{J}\phi _k = f \quad \implies \quad \rho (r) = \sqrt{\frac{1}{k}\int _0^r 2 s f(s)\,\mathrm{d}s}; \end{aligned}$$

hence, for the equation $\mathrm{J}\phi _k=f$ to be solvable for some $k\in {\mathbb {Z}}\backslash \{0\}$, one of the following conditions ought to hold:

2.6 $$\begin{aligned}&\int _0^r 2 s f(s)\,\mathrm{d}s\le 0 \quad \text{ for } \text{ a.e. } r, \end{aligned}$$

2.7 $$\begin{aligned}&\int _0^r 2 s f(s)\,\mathrm{d}s\ge 0 \quad \text{ for } \text{ a.e. } r. \end{aligned}$$

Conversely, whenever *f* satisfies (2.6)–(2.7), we will take $\rho$ as in (2.5), so that $\phi _k$ is a *formal* solution of $\mathrm{J}\phi _k =f$. Indeed, note that (2.6)–(2.7) are not enough to ensure the existence of generalised radial stretching solutions with the required regularity.

### Example 2.5

If $f=1_{\mathbb A(1,2)}$ then, for any $k\in {\mathbb {N}}\backslash \{0\}$, the map $\phi _k$ is in $\bigcup _{1\le q <2} W^{1,q}\backslash W^{1,2}(B_2,{\mathbb {R}}^2)$. Indeed, according to (2.5), we have $\rho (r)=0$ if $r\in [0,1]$ while

$$\begin{aligned} \rho (r)=\sqrt{\frac{r^2-1}{k}} \quad \text{ if } r\in [1,2] \end{aligned}$$

for some $k\in {\mathbb {N}}$. A simple calculation then shows that

$$\begin{aligned} \int _1^{1+\delta } |{{\dot{\rho }}}(r)|^2 r \,\mathrm{d}r=\int _1^{1+\delta } \frac{r^3}{k(r-1)(r+1)} \,\mathrm{d}r=+\infty \end{aligned}$$

for any $\delta >0$ and the claim follows from Lemma 2.4.

Our next goal is to give a condition on *f* which ensures that $\phi _k$ is in ${\dot{W}}^{1,2p}$. We begin by rewriting the quantity $\lambda [f]$, defined in (1.7), in terms of $\phi _1$.

### Lemma 2.6

Suppose $f\in L^1(B)$ satisfies (2.7) a.e. and let $\rho$ be defined as in (1.6). Then

2.8 $$\begin{aligned} |{{\dot{\rho }}}(r)|\le \lambda [f] \frac{\rho (r)}{r}. \end{aligned}$$

### Proof

The proof is an elementary calculation: according to the definition of $\rho$,

$$\begin{aligned} {{\dot{\rho }}}(r)= \frac{r f(r)}{\sqrt{\int _0^r 2 s f(s) \,\mathrm{d}s}} \quad \implies \quad |{{\dot{\rho }}}(r)| \frac{r}{\rho (r)} = \frac{r^2 |f(r)|}{\int _0^r 2 s f(s) \,\mathrm{d}s} =\frac{|f(r)|}{\fint _{B_r(0)} f \,\mathrm{d}x}. \end{aligned}$$

As $\lambda [f]$ is the essential supremum of the right-hand side, the conclusion follows. $\quad \square$

We now prove the following variant of Proposition A:

### Proposition 2.7

Let $f \in {\mathscr {H}}^p({\mathbb {R}}^2)$ be radially symmetric and satisfy (2.7). Then

$$\begin{aligned} \Vert \mathrm{D}\phi _1\Vert ^2_{L^{2p}({\mathbb {R}}^2)} \lesssim (1+\lambda _\infty [f]^2) \,\Vert f\Vert _{{\mathscr {H}}^p({\mathbb {R}}^2)}, \end{aligned}$$

where we define $\lambda _\infty [f]\equiv \lim _{R\rightarrow \infty } \lambda _R[f]$ and $\lambda _R$ is defined in (1.8).

For other related results see [37, 50, §3] and [59, §7].

### Proof

By rescaling (2.8) and combining it with Lemma 2.4, we get

2.9 $$\begin{aligned} \begin{aligned} \Vert \mathrm{D}\phi _1 \Vert _{L^{2p}(B_R(0))}^{2p}&\approx \int _0^R \left( |{{\dot{\rho }}}(r)|^{2p} + \left| \frac{\rho (r)}{r}\right| ^{2p} \right) r \,\mathrm{d}r \\&\le (1+\lambda _R[f]^{2p})\int _0^R \left| \frac{\rho (r)}{r}\right| ^{2p} r \,\mathrm{d}r\\&= (1+\lambda _R[f]^{2p})\int _{B_R(0)}\left| \fint _{B_{|x|}(0)} f \,\mathrm{d}y\right| ^p \,\mathrm{d}x. \end{aligned} \end{aligned}$$

We denote by *M* be the (non-centred) Hardy–Littlewood maximal function. If $p>1$, we may use the maximal inequality and send $R\rightarrow \infty$ in (2.9) to get

$$\begin{aligned} \Vert \mathrm{D}\phi _1 \Vert _{L^{2p}({\mathbb {R}}^2)}^{2p} \le (1+\lambda _\infty [f]^{2p})\int _{{\mathbb {R}}^2} \left| M f(x)\right| ^p \,\mathrm{d}x\lesssim (1+\lambda _\infty [f]^{2p}) \int _{{\mathbb {R}}^2} |f(x)|^p \,\mathrm{d}x; \end{aligned}$$

it now suffices to apply the elementary inequality $(1+t^{2p})^{1/p}\le 1+t^2$, valid for $t\ge 0$.

In the case $p=1$ we need to argue in a more careful way. We use the fact that

$$\begin{aligned} \Vert f(|r|) \Vert _{{\mathscr {H}}^1({\mathbb {R}},|r|\,\mathrm{d}r)} \approx \Vert f(|x|) \Vert _{{\mathscr {H}}^1({\mathbb {R}}^2,\,\mathrm{d}x)}, \end{aligned}$$

see the proof of [20, Corollary (2.27)]. Recall that an ${\mathscr {H}}^1({\mathbb {R}}, |r|\,\mathrm{d}r)$-atom is simply a function $a:{\mathbb {R}}\rightarrow {\mathbb {R}}$ such that

$$\begin{aligned} \text {supp}\,a\subset [r_1,r_2], \qquad \Vert a \Vert _\infty \le \frac{1}{\int _{r_1}^{r_2} |s| \,\mathrm{d}s}, \qquad \int _{\mathbb {R}}a(r) |r| \,\mathrm{d}r=0, \end{aligned}$$

for some real numbers $r_1<r_2$, and that moreover for any $f\in {\mathscr {H}}^1({\mathbb {R}},|r|\,\mathrm{d}r)$ there exist atoms $a_i$ and real numbers $c_i\in {\mathbb {R}}$ such that

2.10 $$\begin{aligned} 0=\lim _{N \rightarrow \infty } \left\| f - \sum _{i=1}^N c_i a_i \right\| _{{\mathscr {H}}^1({\mathbb {R}}, |r| \,\mathrm{d}r)}, \qquad \sum _{i=1}^\infty |c_i| \lesssim \Vert f \Vert _{{\mathscr {H}}^1({\mathbb {R}},|r| \,\mathrm{d}r)}. \end{aligned}$$

Sending $R\rightarrow \infty$ in (2.9), we can estimate

$$\begin{aligned} \Vert \mathrm{D}\phi _1 \Vert _{L^{2}({\mathbb {R}}^2)}^{2}&\le (1+\lambda _\infty [f]^{2}) \int _{{\mathbb {R}}^2}\left| \fint _{B_{|x|}(0)} f \,\mathrm{d}y\right| \,\mathrm{d}x\\&= (1+\lambda _\infty [f]^{2}) \int _0^\infty \frac{1}{r} \int _0^r 2 \, f(s) s \,\mathrm{d}s\,\mathrm{d}r \\&= (1+\lambda _\infty [f]^{2})\lim _{\varepsilon \rightarrow 0} \int _\varepsilon ^{1/\varepsilon } \frac{1}{r} \int _{-r}^r f(s)|s|\,\mathrm{d}s\,\mathrm{d}r, \end{aligned}$$

where we also used $f=f(|r|)$ in the last equality.

Fix $\varepsilon > 0$. By using (2.10) and the dominated convergence theorem,

$$\begin{aligned} \int _\varepsilon ^{1/\varepsilon } \frac{1}{r} \int _{-r}^r f(s)|s|\,\mathrm{d}s\,\mathrm{d}r = \lim _{N \rightarrow \infty } \sum _{j=1}^N c_j \int _\varepsilon ^{1/\varepsilon } \frac{1}{r} \int _{-r}^r a_j(s)|s|\,\mathrm{d}s\,\mathrm{d}r. \end{aligned}$$

When $N \in {\mathbb {N}}$, suppose *a* is one of the atoms $a_1,\ldots ,a_N$ and let $0\le {{\tilde{r}}}_1< {{\tilde{r}}}_2$ be, respectively, the minimum and the maximum of $|\cdot |$ over $[r_1,r_2]$. Then

$$\begin{aligned} \int _\varepsilon ^{1/\varepsilon } \frac{1}{r} \int _{-r}^r a(s)|s|\,\mathrm{d}s\,\mathrm{d}r&= \int _{\max \{\varepsilon ,{{\tilde{r}}}_1\}}^{\min \{1/\varepsilon ,{{\tilde{r}}}_2\}} \frac{1}{r} \int _{-r}^r a(s)|s|\,\mathrm{d}s\,\mathrm{d}r \\&\le \frac{\int _{{{\tilde{r}}}_1}^{{{\tilde{r}}}_2} \frac{1}{r} \int _{-r}^r|s|\,\mathrm{d}s\,\mathrm{d}r}{\int _{r_1}^{r_2} |s| \,\mathrm{d}s} = \frac{{{\tilde{r}}}_2^2-{{\tilde{r}}}_1^2}{2\int _{r_1}^{r_2} |s| \,\mathrm{d}s}\le 1. \end{aligned}$$

By first letting $N \rightarrow \infty$ and then $\varepsilon \rightarrow 0$, we see that the conclusion follows from (2.10). $\quad \square$

Note that, in Proposition 2.7, one cannot hope for an estimate which does not depend on $\lambda [f]$: this is easily seen by considering data of the type $f(r)=\varepsilon 1_{B_1(0)} + 1_{\mathbb A(1,2)}$ for $r\in [0,2]$ and performing calculations identical to those in Example 2.5, c.f. also (4.5).

Finally, Proposition 2.7 easily implies Proposition A:

### Proof of Proposition A

Let us extend *f* by zero outside *B*. If $p>1$, applying Proposition 2.7 the conclusion follows. To deal with the case $p=1$, we recall that

$$\begin{aligned} \Vert f \Vert _{L\log L(B)} \approx \Vert Mf\Vert _{L^1(B)}, \end{aligned}$$

whenever $\text {supp}\,f\subset {\overline{B}}$; see for instance [57, page 23]. Since $f=0$ outside ${{\overline{B}}}$, we take $R=1$ and apply (2.9), which is valid for any $p\in [1,\infty )$, to see that

$$\begin{aligned} \Vert \mathrm{D}\phi _1\Vert _{L^2(B)}^2\lesssim (1+\lambda [f]^2) \Vert M f\Vert _{L^1(B)}, \end{aligned}$$

proving the desired conclusion.

The last claim in Proposition A is not difficult to prove and we refer the reader to [37] for further details.

$$\square $$

To conclude this section we prove the claim made in Remark 1.5 and we show that energy minimisers are unique if and only if they are symmetric. Arguments similar to the ones used in the proof will be useful in Sects. 3 and 5.

### Lemma 2.8

A 2-energy minimiser $u\in W^{1,2}_\text{ id }(B,B)$ for $f\in X^1(B)$ commutes with rotations, i.e.,

$$\begin{aligned} e^{ i \alpha } u= u(e^{i \alpha } \cdot ) \quad \text { for all } \alpha \in [0,2\pi ], \end{aligned}$$

if and only if $u=\phi _1$.

### Proof

If *u* commutes with rotations then $u(\mathbb S_r)= \mathbb S_{\rho (r)}$, for some $\rho (r)\ge 0$. As *u* is necessarily a homeomorphism, $\rho$ is strictly increasing in *r*. It follows that there is $k_r\in C^0([0,2\pi ])$ such that

$$\begin{aligned} u(r e^{ i \theta }) = \rho (r) e^{i k_r(\theta )}. \end{aligned}$$

As *u* commutes with rotations, we see that $k_r(\theta + \alpha )-(k_r(\theta )+\alpha ) \in 2\pi {\mathbb {Z}}$ for all $\alpha \in [0,2\pi ].$ In fact, by continuity in $\alpha$ and $\theta$,

2.11 $$\begin{aligned} k_r(\theta + \alpha )-(k_r(\theta )+\alpha )=2\pi l \end{aligned}$$

for some $l\in {\mathbb {Z}}$. By taking two (distributional) derivatives of (2.11) in $\alpha$, we deduce that $k_r(\theta )=\theta + c_r$. Due to the regularity of *u*, $c_r$ is also a $W^{1,2}$ function in *f*. As *u* is an energy minimiser, (2.4) shows that $c_r$ ought to be constant, and thus $c_r=0$ by the boundary condition. Hence $u=\phi _1$.

$$\square $$

## A Class of Data with Symmetric Energy Minimisers

The purpose of this section is to prove a more general version of Theorem B(i). The key step in doing so is the following proposition, which may be of independent interest:

### Proposition 3.1

Let $p\in [1,\infty )$ and $f\in {\mathscr {H}}^p({\mathbb {R}}^2)$ be a radially symmetric function such that, for some $\varLambda \ge 1$ and a.e. $r\in (0,+\infty )$,

3.1 $$\begin{aligned} | f(r)|\le \varLambda \fint _{B_r(0)} f \,\mathrm{d}x. \end{aligned}$$

Let $\phi _1$ denote the radial stretching solving $\mathrm{J}\phi _1=f$, as in (1.6).

(i) Let $u\in W^{1,2}_\text{ loc }({\mathbb {R}}^2, {\mathbb {R}}^2)$ be a solution of $\mathrm{J}u =f$ such that, for a.e. ${r\in (0,+\infty )}$,
  3.2 $$\begin{aligned} 4 \pi \int _{B_r} \mathrm{J}u \,\mathrm{d}x \le \biggr (\int _{0}^{2\pi }\bigg |\frac{\partial _\theta u}{r}\bigg |(r e^{i \theta })\,\mathrm{d}\theta \biggr )^2. \end{aligned}$$
  Then, with *Z* denoting the Zhukovsky function $Z(\varLambda )\equiv \frac{1}{2} \left( \frac{1}{\varLambda }+ \varLambda \right)$, we have the estimate
  3.3 $$\begin{aligned} \int _{0}^{2\pi } |\mathrm{D}\phi _1|^{2p}(r e^{i \theta }) \,\mathrm{d}\theta \le Z(\varLambda )\int _{0}^{2\pi } |\mathrm{D}u|^{2p}(r e^{i \theta }) \,\mathrm{d}\theta \end{aligned}$$
  for a.e. $$r\in (0,\infty ).$$
(ii) Suppose $\varLambda = 1$. For a.e. $r\in (0,\infty )$, we have that
  $$\begin{aligned} (3.3)\text { holds with equality} \iff {\left\{ \begin{array}{ll} (3.2)\text { holds with equality,}\\ \partial _r u \bot \partial _\theta u \text { in } \mathbb S_r,\\ \partial _\theta u \text { is constant in } \mathbb S_r. \end{array}\right. } \end{aligned}$$

In the statement of Proposition 3.1, as well as in its proof, *u* denotes the precise representative of the equivalence class $[u]\in W^{1,2p}_\text{ loc }$. We refer the reader to [28] for the definition and properties of precise representatives.

We note that condition (3.2) is a *parametric version of the isoperimetric inequality*. In particular, it holds under natural assumptions including the setting of Theorem B, see already Proposition 3.3 below. It is also worth mentioning that, in (3.1), we make implicitly a *choice of orientation*. Indeed, in order to ensure the existence of generalised radial stretchings solving the equation, it must be the case that the function $r\mapsto \int _{B_r} f \,\mathrm{d}x$ does not change sign, see (2.6)–(2.7). Clearly (3.1) implies that this function is non-negative. There is an analogue of Proposition 3.1 in the case where $\int _{B_r} f \,\mathrm{d}x$ is always non-positive: in that case, we replace $\phi _1$ with $\phi _{-1}$.

The proof of Proposition 3.1 relies on a convexity argument. However, before proceeding with it, we record the following elementary lemma:

### Lemma 3.2

Define $\psi :(0,\infty )\times {\mathbb {R}}\rightarrow {\mathbb {R}}$ by $\psi (a,b)\equiv a+b^2/a$. Then

(i) for each $b\in {\mathbb {R}}$, the function $\psi (\cdot , b)$ is convex;
(ii) for each $b\in {\mathbb {R}}$, the function $a\mapsto \psi (a,b)$ is decreasing in (0, |*b*|) and increasing in $(|b|,+\infty )$ and it has a global minimum at $a=|b|$;
(iii) for $\varLambda >0$, if $a_2\le a_1$ and $|b|\le \varLambda a_2$ then $\psi (a_2,b)\le Z(\varLambda ) \psi (a_1,b)$.

In fact, the function $\psi :(0,\infty )\times {\mathbb {R}}\rightarrow {\mathbb {R}}$ is convex, although this will not be needed.

### Proof

The first two properties are readily checked. To prove (iii), note that when $|b|\le a_2$ the conclusion follows from (ii), since $1\le Z(\varLambda )$. When $a_2< |b|$ then, by applying (ii) twice,

$$\begin{aligned} \psi (a_2,b)\le \psi (b/\varLambda ,b)=Z(\varLambda ) \psi (b,b) \le Z(\varLambda )\psi (a_1,b). \end{aligned}$$

$$\square $$

### Proof of Proposition 3.1

We first deal with the case $p=1$. Note that $\phi _1$ is continuous and denote also by *u* the precise representative of the class $[u]\in W^{1,2}_\text{ loc }$. Consider the set of “good” radii

$$\begin{aligned} \mathcal G \equiv \biggr \{r\in (0,\infty ): \begin{array}{l} u|_{\mathbb S_r} \text{ is } \text{ absolutely } \text{ continuous, } (3.1) \text{ and } (3.2) \text{ hold, } \\ \text{ and } \mathrm{J}u(x) = f(x) \text{ for } {\mathscr {H}}^1\text{-a.e. } x\in \mathbb S_r \end{array} \biggr \}. \end{aligned}$$

Since *u* is a Sobolev function, our hypotheses together with an application of Fubini’s theorem show that the $\mathcal G$ has full measure, i.e. ${\mathscr {L}}^1({\mathbb {R}}^+\backslash \mathcal G)=0$.

Fix $r\in \mathcal G$. The crucial observation is that $\phi _1$ satisfies the isoperimetric inequality (3.2) with equality: this is easily checked directly from (1.6), but it can also be seen as a consequence of the fact that $\phi _1$ maps circles to circles and has degree one. Hence, as *u* satisfies (3.2), by assumption,

$$\begin{aligned} \left( \int _{0}^{2\pi } \bigg |\frac{\partial _\theta \phi _1}{r}\bigg |(r e^{i \theta })\,\mathrm{d}\theta \right) ^2 = 4 \pi \int _{B_r} f \,\mathrm{d}x \le \biggr (\int _{0}^{2\pi } \bigg |\frac{\partial _\theta u}{r}\bigg |(r e^{i \theta })\,\mathrm{d}\theta \biggr )^2. \end{aligned}$$

Moreover, $\mathrm{D}\phi _1(re^{i \theta })$ is constant on $\mathbb S_r$ and so, using Jensen’s inequality, we arrive at

3.4 $$\begin{aligned} \bigg |\frac{\partial _\theta \phi _1}{r}\bigg |^2(re^{i \theta }) = \left( \fint _{0}^{2\pi } \bigg |\frac{\partial _\theta \phi _1}{r}\bigg |(re^{i \theta }) \,\mathrm{d}\theta \right) ^2 \le \fint _{0}^{2\pi } \bigg |\frac{\partial _\theta u}{r}\bigg |^2(re^{i \theta })\,\mathrm{d}\theta . \end{aligned}$$

We note the following cofactor identity: if $\nu \in \mathbb S^{1}$ and $A\in {\mathbb {R}}^{2\times 2}$,

$$\begin{aligned} \det A = \det A \langle \nu , \nu \rangle = \langle \text{ cof }(A)^\text{ T } A \nu , \nu \rangle = \langle A \nu , \text{ cof }(A) \nu \rangle . \end{aligned}$$

Using the Cauchy–Schwarz inequality and the fact that $|\text{ cof }(A)\nu |=|A\nu ^\bot |$, we have

3.5 $$\begin{aligned}&\det A \le |A\nu | |\text{ cof }(A) \nu | \nonumber \\&\qquad \implies |A\nu ^\bot |^2 +\frac{(\det A)^2}{|A\nu ^\bot |^2}\le |A\nu ^\bot |^2+ |A\nu |^2= |A|^2.\nonumber \\ \end{aligned}$$

We apply (3.5) to $A=\mathrm{D}u(x)$, choosing $\nu =x/r$: since $\mathrm{J}u=f$, $\mathrm{D}u(x)\cdot \nu =\partial _r u(x)$ and $\mathrm{D}u(x)\cdot \nu ^\bot =\partial _\theta u(x)/r$, we get

3.6 $$\begin{aligned} \begin{aligned} \fint _{0}^{2\pi }\psi \bigg (\frac{|\partial _\theta u(r e^{i \theta })|^2}{r^2}, f(r)\bigg )\,\mathrm{d}\theta&= \fint _{{\mathbb {S}}_r} \frac{|\partial _\theta u(r e^{i \theta })|^2}{r^2} + \frac{ r^2f(r)^2}{ |\partial _\theta u(r e^{i \theta })|^2} \,\mathrm{d}\theta \\&\le \fint _{0}^{2\pi } |\mathrm{D}u|^2(r e^{i \theta })\,\mathrm{d}\theta , \end{aligned} \end{aligned}$$

where $\psi$ is as in Lemma 3.2. By Lemma 3.2(i), Jensen’s inequality applies to yield

3.7 $$\begin{aligned} \psi \left( \fint _{0}^{2\pi } \frac{|\partial _\theta u(r e^{i \theta })|^2}{r^2}\,\mathrm{d}\theta , f(r)\right) \le \fint _{0}^{2\pi }\psi \bigg (\frac{|\partial _\theta u(r e^{i \theta })|^2}{r^2},f(r)\bigg ) \,\mathrm{d}\theta . \end{aligned}$$

We now take

$$\begin{aligned} a_1= & {} \fint _{0}^{2\pi }\frac{|\partial _\theta u(r e^{i \theta })|^2}{r^2}\,\mathrm{d}\theta , \quad a_2=\frac{|\partial _\theta \phi _1(r e^{i \theta })|^2}{r^2}\\= & {} \frac{\rho (r)^2}{r^2} ,\quad b=f(r)=\frac{\rho (r) {{\dot{\rho }}}(r)}{r}. \end{aligned}$$

From (3.4) we have that $a_2\le a_1$ and from (2.8) we have $|b|\le \varLambda a_2$. Hence Lemma 3.2(iii), combined with (3.6) and (3.7), gives

$$\begin{aligned} \psi \left( \frac{|\partial _\theta u(r e^{i \theta })|^2}{r^2}, f(r)\right)&\le Z(\varLambda )\, \psi \left( \fint _{0}^{2\pi } \frac{|\partial _\theta u(r e^{i \theta })|^2}{r^2}\,\mathrm{d}\theta , f(r)\right) \\&\le Z(\varLambda ) \fint _{0}^{2\pi } |\mathrm{D}u|^2(r e^{i \theta })\,\mathrm{d}\theta . \end{aligned}$$

One can verify directly that $\phi _1$ satisfies (3.6) with equality, but see also the proof of part (ii) below for a more detailed justification; thus

$$\begin{aligned} \fint _{0}^{2\pi } |\mathrm{D}\phi _1|^2(r e^{i \theta })\,\mathrm{d}\theta = |\mathrm{D}\phi _1|^2(r e^{i \theta })= \psi \left( \frac{|\partial _\theta \phi _1(r e^{i \theta })|^2}{r^2}, f(r)\right) . \end{aligned}$$

This proves (3.3) when $p=1$.

The case $p>1$ follows from the case $p=1$: since $t\mapsto t^{2p}$ is a strictly convex, increasing function over ${\mathbb {R}}^+$, we can apply Jensen’s inequality to conclude that

$$\begin{aligned} \fint _{0}^{2\pi } |\mathrm{D}\phi _1|^{2p}(r e^{i \theta })\,\mathrm{d}\theta&= \left( \fint _0^{2\pi }|\mathrm{D}\phi _1|^2(r e^{i \theta }) \,\mathrm{d}\theta \right) ^{p} \\&\le \left( \fint _0^{2\pi }|\mathrm{D}u|^2(r e^{i \theta })\,\mathrm{d}\theta \right) ^{p}\le \fint _{0}^{2\pi } |\mathrm{D}u|^{2p}(r e^{i \theta })\,\mathrm{d}\theta , \end{aligned}$$

where we also used the fact that $\mathrm{D}\phi _1$ is constant in $\mathbb S_r$ in the first equality.

It remains to prove (ii), which characterises the equality cases in (3.3). This will follow by inspection of the previous proof. Firstly, according to Lemma 3.2(ii), $\psi (a_2,b)<\psi (a_1,b)$ if $b\le a_2<a_1$. Thus, to have equality in (3.3), we must have $a_2=a_1$, that is, we must also have equality in (3.2). Secondly, we must have equality in (3.4), which holds if and only if $\theta \mapsto |\partial _\theta u|(r e^{i \theta })$ is constant, as the function $|\cdot |^2$ is strictly convex. Finally, we must also have equality in (3.5); by the equality cases in Cauchy–Schwarz, equality holds if and only if $\text{ cof }(\mathrm{D}u)\nu$ is parallel to $\mathrm{D}u\cdot \nu$, or equivalently if and only if $\partial _r u \bot \partial _\theta u$; this is the case, in particular, when *u* is a radial stretching^2^, c.f. (1.9). To conclude, note that we also have equality in (3.6) whenever we have equality in (3.5). This completes the proof.

$$\square $$

We next show that (3.2) holds under natural assumptions.

### Proposition 3.3

Fix $p\in [1,\infty )$ and $R>0$. Let $u\in W^{1,2p}(B_R(0),{\mathbb {R}}^2)$ be a continuous map. Suppose furthermore that for a.e. $r\in (0,R)$ the change of variables formula

3.8 $$\begin{aligned} \int _{B_r} \mathrm{J}u \,\mathrm{d}x = \int _{{\mathbb {R}}^2} \mathrm{deg}(y,u,B_r) \,\mathrm{d}y \end{aligned}$$

holds. Then (3.2) holds for a.e. $r\in (0,R)$. Moreover, equality holds in (3.2) if and only if $u(\mathbb S_r)$ is a circle which is traversed one time.

In (3.8), $\text{ deg }(y,u,B_r)$ denotes the *topological degree* of *u* at *y* with respect to $B_r$. We note that, in the context of Theorem B(i), $\text{ deg }(y,u,B_r)=1$ always, as solutions are automatically homeomorphisms; under this assumption, the proof of Proposition 3.3 is somewhat simpler. Nonetheless, the statement for general maps given in Proposition 3.3 is required to prove Corollary C.

### Proof

We first note that due to the Sobolev regularity of *u*, $u(\mathbb S_r)$ is a continuous rectifiable curve for almost every $r\in (0,R)$ and hence we may restrict to such *r* without loss of generality. We now recall the following *generalised isoperimetric inequality*. Given a continuous rectifiable curve $\varGamma$, let $(E_k)_k$ be the components of ${\mathbb {R}}^2\backslash \varGamma$; on each $E_k$, $\varGamma$ has a well-defined winding number $w_k$. Then we have

3.9 $$\begin{aligned} 4\pi \sum _k w_k^2\, {\mathscr {L}}^2(E_k) \le l(\varGamma )^2 \end{aligned}$$

with equality if and only if $\varGamma$ is a circle traversed a finite number of times in a given direction. Here $l(\varGamma )$ denotes the length of $\varGamma$. This inequality was proved implicitly in [29, p. 487] and then later in [5], but see also [55] for a comprehensive overview.

We want to apply (3.9) when $\varGamma :\mathbb S^1\rightarrow {\mathbb {R}}^2$ is the curve $\varGamma (\theta )=u(re^{i \theta })$. Recall that, at a point *y*, the winding number of the curve $\varGamma$ with respect to *y* is just $\text{ deg }(y,u,B_r)$, see for instance [25, §6.6]. Since $l(\varGamma )= \int _{\mathbb S_r} |\text{ cof }(\mathrm{D}u)\nu |\,\mathrm{d}\theta$, we can use (3.9) to get

$$\begin{aligned} \int _{{\mathbb {R}}^2} \text{ deg }(y,u,B_r)^2 \,\mathrm{d}y\le \frac{1}{4\pi } \biggr (\int _{\mathbb S_r} |\text{ cof }(\mathrm{D}u)\,\nu |\,\mathrm{d}\theta \biggr )^2. \end{aligned}$$

As the topological degree is an integer, we deduce from (3.8) that

3.10 $$\begin{aligned} \int _{B_r} \mathrm{J}u\,\mathrm{d}x\le \frac{1}{4\pi } \biggr (\int _{\mathbb S_r} |\text{ cof }(\mathrm{D}u)\,\nu |\,\mathrm{d}\theta \biggr )^2. \end{aligned}$$

This proves (3.2), since $|\text{ cof }(A)\nu |=|A\nu ^\bot |$ for $A\in {\mathbb {R}}^{2\times 2}$.

The equality cases follow from the equality cases for (3.9) together with the fact that we must have $\text{ deg }(y,u,B_r)=\pm 1$ for $y\in u(B_r)$ to get equality in (3.10). $\quad \square$

### Remark 3.4

For $p>1$ not only is the continuity assumption in Proposition 3.3 not restrictive, but moreover (3.8) also holds automatically, as maps in a supercritical Sobolev space always satisfy the Lusin (N) property. We refer the reader to [33, 38] for further details.

For $p=1$ it is not in general the case that solutions are continuous and satisfy (3.8). However, both properties are satisfied over open sets where $f>0$ a.e., as in this case solutions have finite distortion. Assuming a positive answer to Question 1.6, one can always find solutions satisfying both properties over bounded domains where $f\ge 0$ a.e. [36, Theorem C].

We conclude this section by showing how Theorem B(i) follows from Proposition 3.1.

### Proof of Theorem B(i)

Fix $p\in [1,\infty )$. Since (1.5) holds, Proposition 3.3 applies. As *f* satisfies (3.1) with $\varLambda =1$, we conclude from (3.3) that $\phi _1$ is a 2*p*-energy minimiser.

It remains to show that $\phi _1$ is the unique 2*p*-energy minimiser in $W^{1,2p}_\text{ id }(B,B)$. From Proposition 3.1(ii) and the equality case of Proposition 3.3, we see that for any 2*p*-energy minimiser *u* the curve $u(\mathbb S_r)$ is a circle for every $r\in (0,1)$. Let *z*(*r*) be the centre of the circle $u(\mathbb S_r)$. We may write

3.11 $$\begin{aligned} u(r e^{i \theta }) = z(r)+\psi (r) e^{i \gamma (r,\theta )} \end{aligned}$$

for some continuous functions *z*, $\psi$ and $\gamma$. Note that $u(\mathbb S_r)$ is a circle of radius

$$\begin{aligned} \rho (r)=\sqrt{\int _0^r 2 s f(s)\,\mathrm{d}s }, \end{aligned}$$

which is to say that $\psi =\rho$: indeed, by the change of variables formula,

$$\begin{aligned} \pi \,\psi (r)^2 = {\mathscr {L}}^2(u(B_r(0))=\int _{B_r(0)} f \,\mathrm{d}x. \end{aligned}$$

By Proposition 3.1(ii), we must also have $\gamma (r,\theta )=k(r)\theta + \alpha (r)$, where $k(r)\in {\mathbb {Z}}$. Since $\gamma$ is continuous, *k* is continuous as well, and as $k(1)=1$ we see that $k(r)=1$ for all $r\in (0,1]$. We have thereby arrived at the representation $u(r,\theta ) = z(r) + \rho (r) e^{i(\theta + \alpha (r))}$, and our aim is to show that $z = 0$ and $\alpha = 0$.

We next note that $z(r) = \fint _0^{2\pi } u(r e^{i\theta }) \,\mathrm{d}\theta$ for all $r \in (0,1]$, so that $z \in W^{1,2p}(B_1)$. As a consequence, $e^{i\alpha } = (u-z) e^{-i\theta }/\rho \in W^{1,2p}({\mathbb {A}}(\varepsilon ,1))$ for all $\varepsilon \in (0,1)$. Thus, we may write

$$\begin{aligned} \mathrm{J}u(x)= \frac{\partial _\theta u}{r} \wedge \partial _r u (x)= \mathrm{J}\phi _1(x) + \frac{\rho (r)}{r} x^\bot \wedge \dot{z}(r). \end{aligned}$$

Since $f=\mathrm{J}u = \mathrm{J}\phi _1$ and the argument of *x* is arbitrary, it follows that $\dot{z}=0$. Since $z(1)=0$, we see that $z = 0$. The proof is finished by noting that as *u* has minimal energy, Corollary 2.2 implies that $\alpha$ is constant, so that the boundary condition yields $\alpha =0$. $\quad \square$

Small modifications to the above proof show that in fact the stronger statement in Remark 1.3 holds.

### Proof of Remark 1.3

The only thing left to show is the equality case. The proof of Theorem B(i) shows that, for some $\alpha \in [0,2\pi ]$,

$$\begin{aligned} u(r e^{ i \theta }) = z(r) + \rho (r) e^{i( \pm \theta + \alpha )}; \end{aligned}$$

note that $u-z$ must have degree $\pm 1$, by the equality cases in Proposition 3.3. The same argument as in Theorem B(i) also shows that *z* is in fact constant, say $z(r)=z_0$, finishing the proof.$\quad \square$

Note that Corollary C follows immediately by inspection of the proof of Remark 1.3.

In light of Remark 1.3, it would be interesting to know the extent to which the boundary condition impacts the symmetry of energy minimisers and, in particular, whether the condition $\lambda [f]\le 1$ in Theorem B(i) is sharp for symmetry, c.f. Remark 1.4. A model problem in this direction is to consider, for $\varepsilon >0$, the datum $f_\varepsilon (r)\equiv c_\varepsilon r^\varepsilon$, where $c_\varepsilon \equiv \frac{2}{2+\varepsilon }$ is such that $\fint _{B_1} f_\varepsilon \,\mathrm{d}x = 1$. It is easy to see that

$$\begin{aligned} f_\varepsilon (r)= \frac{2+\varepsilon }{2} \fint _{B_r(0)} f \,\mathrm{d}x, \end{aligned}$$

and hence (3.3) shows that, for $\varepsilon \ll 1$, the energy of the corresponding radial stretchings is arbitrarily close to that of any other energy minimiser. However, we do not know whether the corresponding radial stretchings are 2*p*-energy minimisers in $\text{ id }+W^{1,2p}_0(B,B)$.

## Non-symmetric Energy Minimisers

In this section we prove part (ii) of Theorem B. For a point $z=(x,y)\in {\mathbb {R}}^2$, let us write $|z|_1\equiv |x|+|y|$ for its $\ell ^1$-norm and

$$\begin{aligned} Q_r\equiv \{z\in {\mathbb {R}}^2:|z|_1<r\},\qquad \mathbb A_1(r,R)\equiv \{z\in {\mathbb {R}}^2:r<|z|_1<R\} \end{aligned}$$

for the corresponding balls and annuli. The following example, although simple, is useful:

### Example 4.1

(Mapping a ball onto a square) The map

$$\begin{aligned} \eta (x,y)\equiv \frac{r\,\text{ sgn }(x)}{\sqrt{2}}\,{\left\{ \begin{array}{ll} (1,4/\pi \arctan (y/x) &{} \text {if }|y|< |x|,\\ (4/\pi \arctan (x/y),1)&{} \text {if } |y|\ge |x|,\\ \end{array}\right. }, \end{aligned}$$

is bi-Lipschitz and satisfies a.e. $\det \mathrm{D}\eta = 2/\pi$. For any $r>0$, we also have $R\circ \eta (B_r)=Q_r$, where *R* is a rotation by angle $\frac{\pi }{4}$.

**Fig. 1:**
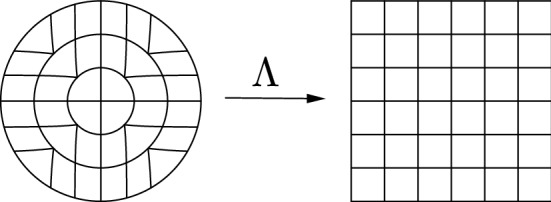
The map from Example 4.1

The map in Example 4.1 can be found in [35]. In fact, Example 4.1 is an explicit particular case of a more general construction, due to Fonseca–Parry [34, Theorem 5.4]. Their result applies to all domains of the following class:

### Definition 4.2

A domain $\varOmega \subset {\mathbb {R}}^n$ is of class ${\mathscr {C}}$ if there are $\varepsilon ,\delta >0$ and $N\in {\mathbb {N}}$ such that:

(i) $B_\varepsilon (0)\subset \varOmega$ and $\varOmega$ is bounded and star-shaped with respect to 0, that is, every ray starting at 0 intersects $\partial \varOmega$ exactly once;
(ii) there is a finite partition $\varOmega =\bigcup _{i=1}^N \varOmega _i$ such that each $\varOmega _i$ is a cone with vertex at 0, $B_\varepsilon (0)\cap \varOmega _i$ is convex, $\partial \varOmega _i\cap \partial \varOmega$ is $C^1$ and satisfies $\nu (x)\cdot x\ge \delta$ for all $x\in \partial \varOmega _i\cap \partial \varOmega$, where $\nu$ denotes the outward unit normal.

Given two domains $\varOmega , {{\tilde{\varOmega }}}$ of class ${\mathscr {C}}$, as they are star-shaped with respect to 0, there is a unique Lipschitz function $\psi :\partial \varOmega \rightarrow (0,+\infty )$ such that $\psi (x) x\in \partial {{\tilde{\varOmega }}}$ for all $x\in \partial \varOmega$. The next theorem was proved in [34], although the statement here is more precise than theirs.

### Theorem 4.3

Let $\varOmega ,{{\tilde{\varOmega }}}$ be two domains of class ${\mathscr {C}}$. Then there is a surjective map $v:\varOmega \rightarrow {{\tilde{\varOmega }}}$ which is *L*-bi-Lipschitz, i.e.

$$\begin{aligned} \frac{1}{L} |x-y|\le |v(x)-v(y)|\le L |x-y| \quad \text{ for } \text{ all } x,y\in {{\overline{\varOmega }}}, \end{aligned}$$

and which solves, for $\psi$ as above,

$$\begin{aligned} {\left\{ \begin{array}{ll} \mathrm{J}v = |{{\tilde{\varOmega }}}|/|\varOmega | &{} \text {in } \varOmega ,\\ v(x)=\psi (x) x &{} \text {for } x\in \partial \varOmega . \end{array}\right. } \end{aligned}$$

Moreover, $L>0$ is a constant which depends only on $\delta ,\varepsilon ,n,N,\text{ diam }(\varOmega )$ and $\text{ diam }({{\tilde{\varOmega }}})$.

Our goal is to use Theorem 4.3 to prove Theorem B(ii). If we do not require *f* to be bounded away from zero, the following yields a simple example:

### Example 4.4

Let $f=\frac{4}{3} 1_{\mathbb A(1,2)}$ and note that the radial stretching $\phi _1$ solving $\mathrm{J}\phi _1=f$ is not in $W^{1,2}(B_{1+\delta }(0))$, for any $\delta >0$, c.f. Example 2.5. Actually, it is a general fact that $W^{1,2}$ solutions of (1.2) cannot be constant in open sets where $f=0$, for otherwise they would have integrable distortion and hence would be open mappings, according to [43].

We can apply Theorem 4.3 to the domains

$$\begin{aligned} \varOmega = \mathbb A(1,2)\cap \{x>0,y>0\},\qquad {{\tilde{\varOmega }}}= B_2(0) \cap \{x>0,y>0\}, \end{aligned}$$

which are star-shaped with respect to (1, 1), to find ${u:\varOmega \rightarrow {{\tilde{\varOmega }}}}$ which is bi-Lipschitz and has constant Jacobian in $\varOmega$. One can then extend *u* to the first quadrant ${B_1(0)\cap \{x>0,y>0\}}$ in a trivial way, using the boundary data on the arc $\mathbb S_1\cap \{x>0,y>0\}$, and then extend *u* to $B_2(0)$ through reflections along the axes, i.e. by setting

**Fig. 2:**
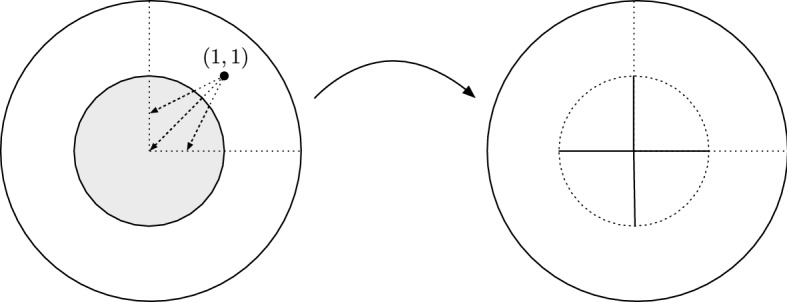
A Lipschitz map which is the identity on $\partial B_2$ and which has $\frac{4}{3} 1_{\mathbb A(1,2)}$ as Jacobian. It maps $\mathbb S_1\cap \{x>0,y>0\}$ onto $\{0\}\times [0,1] \cup [0,1]\times \{0\}$ according to the dotted arrows

4.1 $$\begin{aligned} u(x,y)={\left\{ \begin{array}{ll} (u^1(x,-y), -u^2(x,-y)) &{} \text{ if } x>0, y<0,\\ (-u^1(-x,y), u^2(-x,y)) &{} \text{ if } x<0, y>0,\\ (-u^1(-x,-y), -u^2(-x,-y)) &{} \text{ if } x<0, y<0, \end{array}\right. } \end{aligned}$$

see Fig. 2. Hence there is a Lipschitz solution $u:B_2(0)\rightarrow B_2(0)$ of (1.4) for this data. It follows that for every $p\ge 1$ there is a 2*p*-energy minimiser, but it cannot be symmetric, since the symmetric solution is not even in $W^{1,2}$.

In order to find an example where *f* is bounded away from zero we need a substantially more intricate construction, although the basic idea is similar. We will consider a family of radially symmetric data $f_\varepsilon$ for which the energy of the symmetric solutions $\phi _\varepsilon$ grows unboundedly as $\varepsilon \rightarrow 0$. The key step is to construct solutions of (1.4) for $f_\varepsilon$ with Lipschitz constant uniformly bounded in $\varepsilon$. It then follows that the symmetric solutions cannot be energy minimisers for all data in the family. More precisely, our goal is to prove the following result:

### Theorem 4.5

For $\varepsilon \in [0,1]$, consider the family of data

4.2 $$\begin{aligned} f_\varepsilon \equiv \varepsilon 1_{B_1(0)} + 1_{\mathbb A(1,2)} + \frac{6-\varepsilon }{5} 1_{\mathbb A(2,3)}. \end{aligned}$$

There is a Lipschitz map $u_\varepsilon :B_3(0)\rightarrow B_3(0)$ such that

4.3 $$\begin{aligned} {\left\{ \begin{array}{ll} \mathrm{J}u_\varepsilon = f_\varepsilon &{} \text {in } B_3(0),\\ \mathrm{J}u_\varepsilon = \mathrm{id} &{}\text {on } \mathbb S_3, \end{array}\right. } \end{aligned}$$

and moreover there is a constant *C*, independent of $\varepsilon$, such that

4.4 $$\begin{aligned} \Vert \mathrm{D}u_\varepsilon \Vert _\infty \le C. \end{aligned}$$

Let us just note that, once Theorem 4.5 is proved, the proof of Theorem B is easily finished:

### Proof of Theorem B(ii)

Note that $f_\varepsilon \ge \varepsilon$ and that $\fint _{B_3(0)} f_\varepsilon \,\mathrm{d}x=1$, so that indeed $f_\varepsilon$ satisfies (1.5). Let $\phi _{\varepsilon }$ be the unique radial stretching solving (4.3), where $f_\varepsilon$ is as in (4.2). Explicitly, $\phi _\varepsilon (z)=\rho _\varepsilon (r)\frac{z}{r}$ where, for $r\in (1,2)$, and according to (1.6), we have

4.5 $$\begin{aligned} \rho _\varepsilon (r) = \sqrt{r^2-1+\varepsilon } \quad \implies \quad |\rho '_\varepsilon (r)|^2 =\frac{r^2}{r^2-1+\varepsilon }. \end{aligned}$$

Using Lemma 2.4 we see that, as $\varepsilon \searrow 0$,

$$\begin{aligned} (9\pi )^{\frac{p-1}{2p}}\Vert \mathrm{D}\phi _\varepsilon \Vert _{L^{2p}(B_3)} \ge \Vert \mathrm{D}\phi _\varepsilon \Vert _{L^{2}(B_3)}\nearrow +\infty , \end{aligned}$$

for any $p\in [1,\infty )$. Moreover, by (4.4), the maps $u_\varepsilon$ satisfy

$$\begin{aligned} \Vert \mathrm{D}u_\varepsilon \Vert _{L^{2p}(B_3)}\lesssim 1, \end{aligned}$$

uniformly in $\varepsilon$ and *p*. This completes the proof.

$$\square $$

It thus remains to prove Theorem 4.5. We begin by constructing an auxiliary map.

### Lemma 4.6

(Mapping a wedge onto an ‘A’) For $\varepsilon \in [0,1]$, consider the sets

$$\begin{aligned} \varLambda \equiv \mathbb A_1(2,3)\cap \{y>0\},\qquad A_\varepsilon \equiv \varLambda \cup \{1+\varepsilon (1-|x|)<y\le 2-|x|\}. \end{aligned}$$

Let us write $\partial \varLambda =\varGamma _1\cup \varGamma _2$, where $\varGamma _1\equiv \partial \varLambda \backslash A_\varepsilon$, and consider boundary data

$$\begin{aligned} \gamma _\varepsilon (x,y)= {\left\{ \begin{array}{ll} (x,y) &{} \text {on } \varGamma _1,\\ (x,1+\varepsilon (y-1)) &{} \text {on } \varGamma _2. \end{array}\right. } \end{aligned}$$

There is a surjective Lipschitz map $w_\varepsilon :\varLambda \rightarrow A_\varepsilon$, with $\Vert \mathrm{D}w_\varepsilon \Vert _\infty \le C$, and such that

$$\begin{aligned} {\left\{ \begin{array}{ll} \mathrm{J}w_\varepsilon = \frac{6-\varepsilon }{5} &{} \text {in } \varLambda ,\\ w_\varepsilon =\gamma _\varepsilon &{}\text {on } \partial \varLambda . \end{array}\right. } \end{aligned}$$

### Proof

Take $\varLambda ^+\equiv \varLambda \cap \{x>0\}$ and $A_\varepsilon ^+\equiv A_\varepsilon \cap \{x>0\}$. Consider the map $\tau _\varepsilon \equiv (\tau _\varepsilon ^1,\tau _\varepsilon ^2)$ defined for $(x,y)\in \varLambda ^+$ by

$$\begin{aligned} \tau ^1_\varepsilon (x,y)&\equiv x\\ \tau ^2_\varepsilon (x,y)&\equiv {\left\{ \begin{array}{ll} \frac{1}{2} (2 \varepsilon (x-1) (x+y-3)-x^2+3x+y^2-y) &{} \text{ if } x\in [0,1],\\ \frac{1}{2} (x (2 y-5)+x^2+y^2-3 y +6) &{} \text{ if } x\in [1,2],\\ \frac{1}{2} y (x+y-1) &{} \text{ if } x\in [2,3]. \end{array}\right. } \end{aligned}$$

Since $\mathrm{J}\tau _\varepsilon = \partial _y \tau ^2_\varepsilon$, it follows that, for $(x,y)\in \varLambda ^+$we have

$$\begin{aligned} \mathrm{J}\tau _\varepsilon (x,y)= {\left\{ \begin{array}{ll} \varepsilon (x-1) +y-\frac{1}{2}&{} \text{ if } x\in [0,1],\\ x+y-\frac{3}{2} &{} \text{ if } x\in [1,2],\\ \frac{1}{2}(x-1)+y &{} \text{ if } x\in [2,3]. \end{array}\right. } \end{aligned}$$

**Fig. 3:**
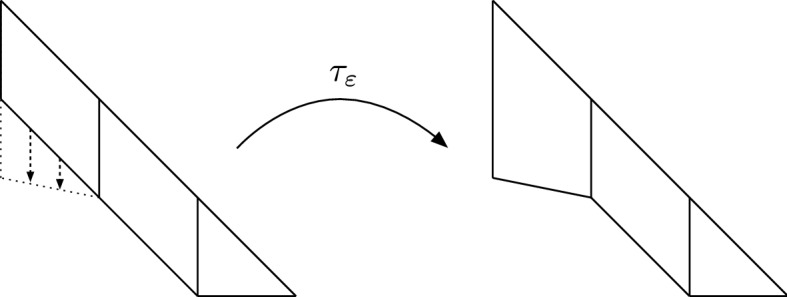
The map $\tau _\varepsilon$, mapping $\varLambda ^+$ onto $A^+_\varepsilon$. Apart from the segment with the two dashed arrows, $\tau _\varepsilon$ is the identity on $\partial \varLambda ^+$

It is easy to check that $\mathrm{J}\tau _\varepsilon$ is Lipschitz in $\overline{\varLambda ^+}$ and $\mathrm{J}\tau _\varepsilon \ge \frac{1}{2}$ in $\varLambda ^+$. Note that ${\tau _\varepsilon :\varLambda ^+\rightarrow A^+_\varepsilon }$ is a bi-Lipschitz homeomorphism such that

$$\begin{aligned} \tau _\varepsilon |_{\partial \varLambda ^+\cap \partial \varLambda }=\gamma _\varepsilon |_{\partial \varLambda ^+\cap \partial \varLambda }\qquad \text{ and } \qquad \tau _\varepsilon (\partial \varLambda ^+\backslash \partial \varLambda )= \partial \varLambda ^+\backslash \partial \varLambda ; \end{aligned}$$

in fact, we found $\tau _\varepsilon$ by looking for maps with these properties such that $\tau _\varepsilon ^2$ is a piecewise second order polynomial in *y*. See also Fig. 3.

We now want to apply the Dacorogna–Moser theory to find a map such that ${w_\varepsilon :\varLambda ^+\rightarrow A^+_\varepsilon }$ with constant Jacobian. However, since $A^+_\varepsilon$ is just Lipschitz this cannot be done directly.^3^ Instead, we use Theorem 4.3 to find a bi-Lipschitz homeomorphism $a_\varepsilon :A^+_\varepsilon \rightarrow B_1(0)$ with constant Jacobian (explicitly, we have $\mathrm{J}a_\varepsilon =\frac{2\pi }{6-\varepsilon }$), and we take a solution of

$$\begin{aligned} {\left\{ \begin{array}{ll} \mathrm{J}\sigma _\varepsilon = g_\varepsilon &{}\text{ in } B_1(0),\\ \sigma _\varepsilon = \text{ id } &{}\text{ on } \mathbb S^1, \end{array}\right. } \qquad g_\varepsilon \equiv \frac{6-\varepsilon }{5} \frac{1}{\mathrm{J}\tau _\varepsilon \circ \tau _\varepsilon ^{-1}\circ a_\varepsilon ^{-1}} \end{aligned}$$

Note that, by the change of variables formula, and writing $\chi _\varepsilon \equiv a_\varepsilon \circ \tau _\varepsilon$,

$$\begin{aligned} \int _{B_1(0)} g_\varepsilon =&\frac{6-\varepsilon }{5} \int _{B_1(0)} \frac{\mathrm{J}\chi _\varepsilon ^{-1}}{\mathrm{J}\tau _\varepsilon \circ \chi _\varepsilon ^{-1} \, \mathrm{J}\chi _\varepsilon ^{-1}} = \frac{6-\varepsilon }{5}\int _{B_1(0)} \frac{\mathrm{J}\chi _\varepsilon \circ \chi _\varepsilon ^{-1}}{\mathrm{J}\tau _\varepsilon \circ \chi _\varepsilon ^{-1}} \mathrm{J}\chi _\varepsilon ^{-1}\\ =&\frac{6-\varepsilon }{5}\int _{\varLambda ^+} \frac{\mathrm{J}\chi _\varepsilon }{\mathrm{J}\tau _\varepsilon } = \frac{6-\varepsilon }{5}\frac{2\pi }{6-\varepsilon } |\varLambda ^+|=|B_1(0)|, \end{aligned}$$

thus $g_\varepsilon$ satisfies the required compatibility condition. For any $\alpha \in (0,1)$, we can additionally suppose that

$$\begin{aligned} \Vert \sigma _\varepsilon -\text{ id }\Vert _{C^{1,\alpha }} \le C\left( \alpha ,\Vert g_\varepsilon \Vert _{C^{0,1}}\right) \Vert g_\varepsilon -1\Vert _{C^{0,\alpha }}\le C(\alpha ), \end{aligned}$$

see [56, Theorem 8]. Here the last inequality follows from the fact that the bi-Lipschitz constants of $a_\varepsilon , \tau _\varepsilon$ are uniformly bounded with $\varepsilon \in [0,1]$, since the geometric parameters of $A_\varepsilon ^+$, according to Definition 4.2, are also bounded. We now take $w_\varepsilon :\varLambda ^+\rightarrow A_\varepsilon ^+$ to be

$$\begin{aligned} w_\varepsilon \equiv a_\varepsilon ^{-1} \circ \sigma _\varepsilon \circ a_\varepsilon \circ \tau _\varepsilon \end{aligned}$$

and then extend $w_\varepsilon$ to $\varLambda \backslash \varLambda ^+$ through a reflection, similarly to (4.1). This yields the required map.

$$\square $$

**Fig. 4:**
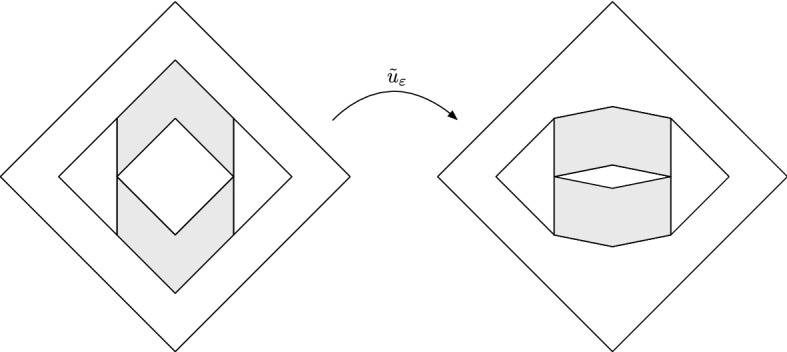
The map ${{\tilde{u}}}_\varepsilon$ constructed in the proof of Theorem 4.5

### Proof of Theorem 4.5

Consider the map $v_\varepsilon$ defined on $Q_2$ by

4.6 $$\begin{aligned} v_\varepsilon (x,y)={\left\{ \begin{array}{ll} (x,\varepsilon y) &{} \text{ if } (x,y)\in Q_1,\\ (x,y) &{} \text{ if } (x,y)\in \mathbb A_1(1,2) \text{ and } |x|>1,\\ (x,y-(1-\varepsilon )(1-|x|)) &{} \text{ if } (x,y)\in \mathbb A_1(1,2) \text{ and } |x|<1, y>0,\\ (x,y+(1-\varepsilon )(1-|x|)) &{} \text{ if } (x,y)\in \mathbb A_1(1,2) \text{ and } |x|<1, y<0. \end{array}\right. } \nonumber \\ \end{aligned}$$

It is easy to check that $\mathrm{J}v_\varepsilon =\varepsilon 1_{Q_1} + 1_{\mathbb A_{1}(1,2)}.$ Let $w_\varepsilon$ be the map from Lemma 4.6 and consider

$$\begin{aligned} {{\tilde{u}}}_\varepsilon \equiv {\left\{ \begin{array}{ll} v_\varepsilon &{} \text{ in } Q_2,\\ w_\varepsilon &{} \text{ in } \varLambda ,\\ {{\bar{w}}}_\varepsilon &{} \text{ in } {{\bar{\varLambda }}}, \end{array}\right. } \qquad \text{ where } {{\bar{\varLambda }}} \equiv \{(x,-y): (x,y)\in \varLambda \} \end{aligned}$$

and ${{\bar{w}}}_\varepsilon (x,y) \equiv (w_\varepsilon ^1(x,-y),-w_\varepsilon ^2(x,-y))$, see Fig. 4. Thus

$$\begin{aligned} \mathrm{J}{{\tilde{u}}}_\varepsilon = \varepsilon 1_{Q_1}+1_{\mathbb A_1(1,2)} + \frac{6-\varepsilon }{5} 1_{\mathbb A_1(2,3)}. \end{aligned}$$

Recall the map $\eta$ from Example 4.1 and let *R* be a rotation by angle $\frac{\pi }{4}$. Taking

$$\begin{aligned} u_\varepsilon \equiv (R \circ \eta )^{-1} \circ {{\tilde{u}}}_\varepsilon \circ (R\circ \eta ), \end{aligned}$$

the proof is finished.

$$\square $$

## Non-uniqueness of Energy Minimisers

The goal of this section is to prove Theorem D, which we restate here.

### Theorem 5.1

Fix $1\le p<\infty$. There exists a radially symmetric function $f\in {\mathscr {H}}^p({\mathbb {R}}^2)$ which has uncountably many 2*p*-energy minimisers, modulo rotations.

A more informative statement can be found in Corollary 5.6, at the end of the section. The proof of Theorem 5.1 relies mostly on elementary tools and the most sophisticated result that we use is the following:

### Theorem 5.2

(Sierpiński) Let $(X_n)$ be disjoint closed sets such that we have ${I=\bigcup _{n\in \mathbb N} X_n}$, where $I=[a,b]\subset {\mathbb {R}}$. There is at most one $n\in {\mathbb {N}}$ such that $X_n$ is non-empty.

Theorem 5.2 is only needed to obtain uncountably many distinct minimisers, as non-uniqueness follows already from more elementary means. We also note that Theorem 5.2 holds more generally for a compact, connected Hausdorff space, see e.g. [26, Theorem 6.1.27]. In the case of an interval there is a simple proof, which we give here for the sake of completeness:

### Proof

Take $Y\equiv \bigcup _n \partial X_n = I\backslash \bigcup _n \text{ int }(X_n)$, which is closed, thus a complete metric space.

We observe that the set *Y* has empty interior in *I*, i.e. any open interval *L* contains an open set *U* disjoint from *Y*. Indeed, from the Baire Category Theorem we see that there is an open set $U\subseteq L$ and some $X_m$ which is dense in *U*. Since $X_m$ is closed, we must have $U\subseteq \text{ int }\,X_m$ and thus *U* is disjoint from *Y*.

By the Baire Category Theorem there is also some open subinterval *J* of *I* and some $n\in {\mathbb {N}}$ such that $\partial X_n$ is dense in $Y\cap J$. Since $\partial X_n$ is closed we have $\partial X_n\cap J = Y\cap J$. Thus $(Y\backslash \partial X_n)\cap J=\emptyset$.

Suppose now that $X_n\ne I$. It follows that *J* intersects $Y\backslash \partial X_n$. Indeed, since *Y* has empty interior in *I*, *J* intersects $I\backslash X_n$ and so it intersects $\text{ int }(X_k)$ for some *k*. Actually, *J* must intersect $\partial X_k$: otherwise, $\text{ int }(X_k)\cap J$ is non-empty, open and closed in *J*, thus $\text{ int }\, X_k=J$, since *J* is connected; clearly this is impossible, since $X_k$ is disjoint from $X_n$. So we proved that *J* intersects $Y\backslash \partial X_n$, contradicting the previous paragraph.

$$\square $$

We are now ready to begin the proof of Theorem 5.1, whose core idea is contained in the following lemma:

### Lemma 5.3

Let *u* be a 2*p*-energy minimiser for a radially symmetric function $f \in {\mathscr {H}}^p({\mathbb {R}}^2)$. For $\alpha _0\in [0,2\pi ]$, consider the set

5.1 $$\begin{aligned} X_{\alpha _0}\equiv \left\{ \alpha \in [0,2\pi ]:u_\alpha = u_{\alpha _0} \text{ modulo } \text{ rotations }\right\} , \quad \text{ where } u_\alpha (z)\equiv u(e^{i \alpha } z). \end{aligned}$$

Assume that $f\in C^0(B_R)$ has a sign. If $X_{\alpha _0}=[0,2\pi ]$ then there is $k\in {\mathbb {Z}}{\setminus }\{0\}$ such that

$$\begin{aligned} u(z)=\phi _k(z) \quad \text{ in } B_R, \text{ modulo } \text{ rotations, } \end{aligned}$$

where $\phi _k$ is as in Definition 2.3.

### Proof

If $X_{\alpha _0}=[0,2\pi ]$ then, for any $\alpha \in [0,2\pi ]$ and $z\in B_R$, we have $|u(e^{i \alpha } z)|= |u(z)|$; that is, circles in $B_R$, centred at zero, are mapped to circles centred at zero.

For each $r\in (0,R)$, we have $0\not \in u(\mathbb S_r)$. Indeed, for each ball $B\Subset B_R$, there is $c=c(B)>0$ such that $f\ge c$ in *B* (or $f\le -c$, but by reversing orientations we can always consider the first case without loss of generality). Thus, in $B_r$, *u* is a map of integrable distortion and so it is both continuous and open [43]. Therefore $\partial (u(B_r))\subseteq u(\partial B_r)=u(\mathbb S_r)$ and we see that $u(\mathbb S_r)\ne \{0\}$. Since $u(\mathbb S_r)$ is a circle, we conclude that $0\not \in u(\mathbb S_r)$.

By Proposition 2.1 we may write

5.2 $$\begin{aligned} u(r,\theta )=\psi (r,\theta )e^{i\gamma (r,\theta )} \end{aligned}$$

where $\psi \in W^{1,2p}([0,R]\times [0,2\pi ])$ and $\gamma \in W^{1,2p}([\varepsilon ,R]\times [0,2\pi ])$ satisfy (2.2) and $\varepsilon >0$ is arbitrary. For $r<R$, $u(\mathbb S_r)=\mathbb S_{r'}$, that is, $\psi (r,\theta )$ is independent of $\theta$. Thus, by (2.3), $\mathrm{J}u =f$ reduces to

5.3 $$\begin{aligned} \partial _r(\psi ^2)\partial _\theta \gamma = 2rf(r), \end{aligned}$$

which is valid for almost every $(r,\theta )\in (0,R]\times [0,2\pi ]$. Since both $\psi$ and the right-hand side are independent of $\theta$ we must have $\gamma (r,\theta )=k\theta + \beta (r)$ and additionally there is the compatibility constraint (2.2) which yields $k\in {\mathbb {Z}}$. We may assume that $k\ne 0$: otherwise (5.3) shows that $f=0$ a.e., which is impossible. Since *u* is a 2*p*-energy minimiser, (2.4) readily implies that $\beta$ is constant. We integrate both sides of (5.3), using $\psi (0)=0$, to find

$$\begin{aligned} \psi (r)^2 =\frac{1}{k} \int _0^r 2s f(s) \,\mathrm{d}s \qquad \text{ for } r<R. \end{aligned}$$

Thus, modulo rotations, $u=\phi _k$ in $B_R$.

$$\square $$

In fact, the same argument applied in an annulus $\mathbb A(R_0,R)$ gives the following variant:

### Lemma 5.4

Consider the setup of Lemma 5.3, but replace $B_R$ by $\mathbb A(R_0,R)$. Then there is $k\in {\mathbb {Z}}{\setminus }\{0\}$ and $c\in {\mathbb {R}}$ such that, in $\mathbb A(R_0,R)$,

$$\begin{aligned} u(z)= \psi (r)e^{2\pi i k \theta } \text{ modulo } \text{ rotations }, \qquad \text{ where } \psi (r)^2 = \frac{1}{k}\int _{R_0}^r 2 s f(s)\,\mathrm{d}s+c. \end{aligned}$$

We now combine the previous two lemmas.

### Lemma 5.5

There is a radially symmetric $f\in {\mathscr {H}}^p({\mathbb {R}}^2)$, admitting a 2*p*-energy minimiser *u*, for which we have $X_0\ne [0,2\pi ]$, where $X_0$ is as in (5.1).

### Proof

We take a function $f:{\mathbb {R}}^2\rightarrow {\mathbb {R}}$ satisfying the following conditions:

5.4 $$\begin{aligned} \begin{aligned}&f\in C^1({\mathbb {R}}^2) \text{ is } \text{ radially } \text{ symmetric },\\&\int _{B_2} f\,\mathrm{d}x = \int _{{\mathbb {R}}^2} f \,\mathrm{d}x = 0\\&f(r) <0 \text{ if } r\in [0,1)\cup (3,4), \quad f(r)>0 \text{ if } r\in (1,3)\cup (4,6),\\&f(r)=[(6-r)^+]^2 \text{ if } r\in (5,+\infty ). \end{aligned} \end{aligned}$$

As *f* decays sufficiently fast near $\mathbb S_6$, we can apply [47, Theorem 4] to see that there is $v\in C^1(\overline{B_6},{\mathbb {R}}^2)$ such that $\mathrm{J}v = f$ and $v=0$ on $\mathbb S_4$; in particular, by extending *v* by zero outside $B_6$, we have $v\in W^{1,2p}({\mathbb {R}}^2,{\mathbb {R}}^2)$. Since the 2*p*-Dirichlet energy is convex, the Direct Method, combined with the sequential weak continuity of the Jacobian, shows that *f* has at least one 2*p*-energy minimiser and we call it *u*, using it to define the sets in (5.1).

Suppose, for the sake of contradiction, that $X_0=[0,2\pi ]$. Using Lemmas 5.3 and 5.4, we deduce that there are angles $\alpha ,\alpha '\in [0,2\pi )$, numbers $k,k'\in {\mathbb {Z}}$ and $c\in {\mathbb {R}}$ such that

$$\begin{aligned} u=e^{i \alpha } \phi _k \text{ in } B_1, \qquad u=e^{i \alpha '} \left( \psi (r) e^{2\pi i k' \theta }\right) \text{ in } \mathbb A(1,3), \end{aligned}$$

where, for $$r\in (1,3)$$,

$$\begin{aligned} \psi (r)^2 =\frac{1}{k'} \int _1^r 2 s f(s) \,\mathrm{d}s + c. \end{aligned}$$

In the notation of Definition 2.3, we must have

$$\begin{aligned} e^{i\alpha +i k \theta } \frac{\rho (1)}{\sqrt{|k|}}\equiv \text{ Tr}_{\mathbb S_1} u_{|B_1}=\text{ Tr}_{\mathbb S_1} u_{|\mathbb A(1,3)} \equiv e^{i\alpha '+i k'\theta } c \end{aligned}$$

in $L^{2p}(\mathbb S_1)$. It is easy to conclude that $\alpha =\alpha '$, $k=k'$ and $c=\rho (1)/\sqrt{|k|}$, and so, modulo rotations, actually $u=\phi _k$ in $B_3$. Arguing as for (4.5), for *f* as in (5.4), we have

$$\begin{aligned} \int _0^3 | {{\dot{\rho }}}(r)|^2 r \,\mathrm{d}r&\gtrsim _f \int _{3/2}^2 \frac{1}{-\int _0^r 2 s f(s) \,\mathrm{d}s} \,\mathrm{d}r\\&= \int _{3/2}^2 \frac{1}{\int _r^2 2sf(s) ds} \,\mathrm{d}r \gtrsim _f \int _{3/2}^2 \frac{1}{4-r^2} \,\mathrm{d}r = +\infty , \end{aligned}$$

and so by Lemma 2.4$u\not \in W^{1,2}(B_3,{\mathbb {R}}^2)$, which is a contradiction. Alternatively, one can infer that $u\not \in W^{1,2}(B_3,{\mathbb {R}}^2)$ from [50, Theorem 3.4].

$$\square $$

### Proof of Theorem 5.1

Let *f* and *u* be as in Lemma 5.5.

For each ${\alpha \in [0,2\pi ]}$, we claim that the set $X_\alpha$ is closed. The case $p>1$ is clear, as *u* is automatically continuous. Indeed, given a sequence $\alpha _j\in X_\alpha$ such that $\alpha _j\rightarrow \alpha _\infty$, we find numbers $\beta _j\in [0,2\pi ]$ such that, for all $z\in {\mathbb {C}}$,

5.5 $$\begin{aligned} u_\alpha (z)=e^{i \beta _j} u_{\alpha _j}(z)=e^{i \beta _j} u(e^{i \alpha _j}z). \end{aligned}$$

By passing to subsequences we can assume that $\beta _j\rightarrow \beta _\infty$ and by continuity of *u* we thus see that $u_{\alpha _\infty }=u(e^{i \alpha _\infty }\cdot )$ is equal to $u_\alpha$, modulo rotations. In the case $p=1$ the argument is similar but slightly more delicate. By the choice of *f*, *u* is necessarily continuous in the *disconnected* open set

5.6 $$\begin{aligned} \varOmega \equiv B_1 \cup \mathbb A(1,3)\cup \mathbb A(3,4) \cup \mathbb A(4,6)\cup \mathbb A(6,+\infty ). \end{aligned}$$

Indeed, continuity in the first four sets in (5.6) follows from the choice of *f*, together with the theory of mappings of finite distortion [38], while continuity in $\mathbb A(6,+\infty )$ follows from [50]: 2-energy minimisers are even Lipschitz continuous in the interior of open sets where $f=0$. We thus see that we can still pass to the limit in (5.5) for all $z\in \varOmega$; as ${\mathbb {R}}^2\backslash \varOmega$ is a null-set, the claim follows.

We may write, for some index set *A*,

$$\begin{aligned}{}[0,2\pi ]= \bigcup _{\alpha \in A} X_\alpha , \qquad \text{ where } \text{ the } \text{ union } \text{ is } \text{ disjoint. } \end{aligned}$$

For distinct $\alpha ,\alpha '\in A$, $X_\alpha$ and $X_{\alpha '}$ correspond to distinct equivalence classes of 2*p*-energy minimisers, where two maps are in the same equivalent class if they are equal up to a rotation. Thus, by Lemma 5.5, we must have $\# A>1$. But now Theorem 5.2 shows that *A* must be uncountable.

$$\square $$

We also note that the proof of Lemma 5.3 yields the following corollary:

### Corollary 5.6

Let $f\in {\mathscr {H}}^p({\mathbb {R}}^2)$ be radially symmetric and suppose *u* is its unique 2*p*-energy minimiser, modulo rotations. If *u* is continuous then $u=\phi _k$ for some $k \in {\mathbb {Z}}{\setminus } \{0\}$.

Clearly the continuity assumption is not restrictive if $p>1$.

### Proof

As in the proof of Lemma 5.3 we conclude that *u* maps circles centred at zero to circles and that $(r,\theta )\mapsto |u(r e^{i \theta })|$ is independent of $\theta$. Thus we write simply |*u*(*r*)|.

We show that the set $\{r \in (0,\infty ) :|u(r)| > 0\}$ is connected. Suppose, by way of contradiction, that there are $r_1< r_2 < r_3$ such that $|u(r_1)|, |u(r_3)| > 0$ but $|u(r_2)| = 0$. We get another 2*p*-energy minimiser for *f* by setting

$$\begin{aligned} v(z) = {\left\{ \begin{array}{ll} u(z), &{} |z| \le r_2, \\ e^{i \pi } u(z), &{} |z| > r_2, \end{array}\right. } \end{aligned}$$

contradicting the assumption that the 2*p*-energy minimiser for *f* is unique modulo rotations.

Thus we can write, for some $0\le R_1 \le R_2\le \infty$,

$$\begin{aligned} \left\{ r \in (0,\infty ) :|u(r)| > 0\right\} =(R_1,R_2). \end{aligned}$$

We can use Lemma 5.4 to conclude that $u=\phi _k$ in $\mathbb A(R_1,R_2)$, modulo rotations. Moreover, clearly we must have $f(r)=0$ if $r\not \in (R_1,R_2)$. Thus $\phi _k(z)=0$ if $r\not \in (R_1,R_2)$ and so $u=\phi _k$ outside $\mathbb A(R_1,R_2)$ as well.

$$\square $$

## Footnotes

1 We believe that the original question in [59] has a misprint and that, in dimensions larger than 2, one should replace the Dirichlet energy considered in [59] by the *n*-harmonic energy, as done in Definition 1.1. Nonetheless, in the planar case we address here, Hélein’s formulation agrees with ours.

2 Although this is not important for our purposes, it is also the case if *u* is conformal.

3 The Dacorogna–Moser theory [23] requires the domain to be at least of class $C^{3,\alpha }$.
